# Adult Nutrient Intakes from Current National Dietary Surveys of European Populations

**DOI:** 10.3390/nu9121288

**Published:** 2017-11-27

**Authors:** Holly L. Rippin, Jayne Hutchinson, Jo Jewell, Joao J. Breda, Janet E. Cade

**Affiliations:** 1 Nutritional Epidemiology Group (NEG), School of Food Science and Nutrition, University of Leeds, Leeds LS2 9JT, UK; J.Hutchinson1@leeds.ac.uk (J.H.); J.E.Cade@leeds.ac.uk (J.E.C.); 2 Division of Noncommunicable Diseases and Promoting Health through the Life-Course, World Health Organization Regional Office for Europe, UN City, Marmorvej 51, DK-2100 Copenhagen, Denmark; jewellj@who.int (J.J.); rodriguesdasilvabred@who.int (J.J.B.)

**Keywords:** national diet surveys, WHO European region, macronutrient intakes, micronutrient intakes, Recommended Nutrient Intakes (RNIs), nutritional epidemiology

## Abstract

The World Health Organization (WHO) encourages countries to undertake national dietary survey (NDS) but implementation and reporting is inconsistent. This paper provides an up-to-date review of adult macro and micronutrient intakes in European populations as reported by NDS. It uses WHO Recommended Nutrient Intakes (RNIs) to assess intake adequacy and highlight areas of concern. NDS information was gathered primarily by internet searches and contacting survey authors and nutrition experts. Survey characteristics and adult intakes by gender/age group were extracted for selected nutrients and weighted means calculated by region. Of the 53 WHO Europe countries, over a third (*n* = 19), mainly Central & Eastern European countries (CEEC), had no identifiable NDS. Energy and nutrient intakes were extracted for 21 (40%) countries but differences in age group, methodology, under-reporting and nutrient composition databases hindered inter-country comparisons. No country met more than 39% WHO RNIs in all age/gender groups; macronutrient RNI achievement was poorer than micronutrient. Overall RNI attainment was slightly worse in CEEC and lower in women and female elderly. Only 40% countries provided adult energy and nutrient intakes. The main gaps lie in CEEC, where unknown nutrient deficiencies may occur. WHO RNI attainment was universally poor for macronutrients, especially for women, the female elderly and CEEC. All countries could be encouraged to report a uniform nutrient set and sub-analyses of nationally representative nutrient intakes.

## 1. Introduction

The burden of malnutrition in the form of overweight and obesity, nutrient deficiency and preventable diet-related non-communicable diseases (NCDs) is significant and worsening [1]. An unhealthy diet is one of the four major behavioral risk factors for NCDs in all WHO regions [2], with the European region proportionately suffering the greatest burden. Here, the four most common NCDs account for 77% of disease and almost 86% premature mortality [1]. The World Health Organization (WHO) European Food and Nutrition Action Plan aims to ‘significantly reduce’ the human, economic and social costs of all forms of malnutrition in the WHO European region [1].

National diet surveys (NDS) have an important role to play in assessing dietary patterns and intakes in populations and informing policy decisions; the WHO European Food & Nutrition Action Plan [1] explicitly encourages member states to ‘strengthen and expand nationally representative diet and nutrition surveys.’ Nutrition and health surveys formed the main source of information for dietary risk factors and physical inactivity in a systematic analysis of disease risk in 21 regions worldwide between 1990–2010 [3]. NDS can help monitor NCDs and malnutrition, identify specific areas of concern, highlight inequalities, guide interventions and evaluate policy impact, thereby ultimately contributing to the promotion of best practice across the region [1]. Imamura et al. [4] evaluated change in global diet patterns over time through either greater consumption of healthy or lesser consumption of unhealthy items and assessed heterogeneity by age, gender, national income and dietary pattern. Higher national income was associated with better diet quality via greater consumption of healthier items but also with higher intake of unhealthy items, demonstrating that socio-economic inequalities persist.

NDS provision across Europe is inconsistent. A recent review found that less than two thirds of countries in WHO Europe have nationally representative NDS and that the majority of gaps lie in Central & Eastern European countries (CEEC) [5]. This is concerning, as nutrition policies in these countries may therefore lack an appropriate evidence base. Novakovic et al. [6] examined selected micronutrient intakes in CEEC compared to other European countries and found that CEEC lacked intake data across all ages. Only 40% of countries in the WHO Europe remit reported adult energy and nutrient intakes from NDS conducted post-2000 and in these, macronutrients were more widely reported than micronutrients [5]. The Global Dietary Database (GDD) houses information on food and nutrient intakes in countries across the world but only includes broad food categories with limited nutrient data and is limited by the inclusion of some regional rather than national data [7].

A comprehensive, updated review of total nutrient intakes across different European populations and subgroups is therefore needed, the results of which could identify where in Europe there is a need to improve diets and whether inequalities exist. This review aims to examine macro and selected micronutrient adult intakes in countries across WHO Europe via the latest NDS for which nutrient intake data is available.

## 2. Materials and Methods

### 2.1. Identifying National Diet Surveys (NDS)

The methods for identifying and accessing NDS have been reported [5]. Briefly, authors of national surveys within WHO Europe were identified using listed contact names and other information from two main reports of NDS [8,9]. Where no response was obtained from authors, further general internet searches were performed on organizations specializing in nutrition to find other potentially useful contact details. Additionally, country responses to WHO questionnaires were mined to obtain relevant references to NDS. Contacts identified were asked to complete a questionnaire to provide information on nationally representative dietary surveys conducted at an individual level since 1990, including links or references to relevant reports. For countries without usable contact details, a systematic database search was performed across Web of Science, Medline and Scopus for nationally representative dietary surveys of adults and children that collected data at an individual level from 1990 to June 2016.

Papers returned were screened for relevance according to the criteria in Table 1. We found 109 nationally representative surveys that collected data on whole diets at an individual level since 1990 across 34 of the 53 countries in the WHO office region; 86 of these included adults. Of these, 78 were conducted since 2000, 60 of which included adults. Further details of all the surveys found are presented in Rippin et al. (in submission) [5].

### 2.2. Data Extracted

Where available, estimated energy and nutrient intake (excluding supplements) by age group and gender was extracted and graphically presented from the latest NDS collected after 2000; for adults, this included surveys from 21 countries. These countries were grouped into regions—Western, Northern and Central & Eastern Europe. For some countries, more recent surveys have been conducted but intake data was not yet available. For example, the Spanish ANIBES survey (2013) did not include micronutrients, so the ENIDE (2011) survey was used instead. Mean intake values were reported by the majority of the 21 countries but where medians were the sole measure of central tendency, these were extracted and used instead. Where energy intakes were given in kcal, these were converted to MJ for consistency across studies.

All macronutrients reported by the 21 countries were included in the data extraction but micronutrients extracted (see Table 2) were limited to those explicitly mentioned in the WHO European Food and Nutrition Action Plan [1] as being currently important to population health in the region. Where possible, WHO nutrient-based guidelines—hereby referred to as Recommended Nutrient Intakes (RNIs)—were used to assess intake adequacy and to highlight areas of concern [10,11,12,13,14], although WHO RNIs for iron are given for different bioavailabilities, so UK Reference Nutrient Intakes (RNIs) were used instead [15]. The RNI for monounsaturated fats (MUFAs) is calculated by the difference between total fat and the sum of saturates (SFA), polyunsaturated fats (PUFA) and trans fats (TFAs), so has not been included. The WHO RNI for free sugars [14] has been adopted as the RNI for added sugars, as no WHO RNI exists for added sugars, yet all surveys that reported sugar in this way used the added rather than free sugar definition. The definition for added sugars is similar but more restrictive to that of free sugars, meaning that free sugar intake would not be overestimated. Depending on the nutrient, the RNIs were variously maximum, minimum or target amounts.

To harmonize data where possible, units of measurement were converted to a common standard unit. Energy intakes and selected nutrients by age group and gender as reported in these latest surveys collected after 2000 were graphed. Omega-3 and omega-6 fatty acids were reported in surveys in various ways, including omega-3, omega-6, linoleic acid and α-linolenic acid in g/day and percentage energy (%E) and eicosapentaenoic acid + docosahexaenoic acid (EPA + DHA) in mg/day. These were converted to grams and %E and grouped into omega-3 and omega-6 fatty acids for clarity. Additionally, mean intakes by age group and gender were weighted by number of individuals surveyed in each group to produce weighted means by country. Regional and overall European weighted means were calculated by multiplying the male/female mean for each country by the latest total national population numbers from 2016 [16], adding this figure for each country and dividing by the total sum of the national populations in each region.

Characteristics of the surveys from the 21 countries were also extracted and reported: these were country name, survey name, year of survey (data collection), dietary methodology, age range and sample size. The percentage WHO RNIs not met by all gender/age groups was recorded. Where reported, surveys presenting nutrient intakes by socio-economic group (SEG) based on social class, income (continuous or grouped) and education level were also noted.

## 3. Results

### 3.1. Data Extracted

Results of NDS coverage across Europe have previously been documented [5]. Adult energy and nutrient intakes (excluding supplements) were extracted from 21 surveys across 21 countries from three regions: five (100%) of Northern European countries (Denmark, Finland, Iceland, Norway, Sweden); 11 (65%) of Western European countries (Andorra, Austria, Belgium, France, Germany, Ireland, Italy, The Netherlands, Portugal, Spain, UK) and five (16%) of CEEC (Estonia, Hungary, Latvia, Lithuania, Turkey). Table 3 shows the characteristics of these surveys. Adult energy and nutrient intakes could not be extracted for 60% (32) of European countries; 19 of these, mainly CEEC, had no identifiable nationally representative survey, making up over a third of WHO Europe countries.

All 21 surveys that reported nutrient information included energy and also carbohydrate, fiber, fat and protein intakes (see Table 4). Most surveys (*n* = 20) included intake data on saturates, MUFAs and PUFAs (Germany did not); however, less than half (*n* = 9) surveys included TFA intakes. The majority of surveys (*n* = 17) included intake levels of sugars, either as total sugars or as added sugars/sucrose; however, Germany, Latvia, Spain and Turkey included neither. Few surveys (*n* = 5) included starch intake data. Half the countries included either omega-3 (*n* = 10) or omega-6 (*n* = 9) fatty acid intakes in some form; eight surveys included both.

All surveys included some micronutrients of interest (see Table 5). Vitamin B12, vitamin D, calcium and iron intakes were reported by all surveys; potassium (not Belgium), folate and sodium (not Italy) were each reported by all but one survey and zinc by all but two (not Belgium and Norway). Iodine was the least reported micronutrient extracted (*n* = 14), though it was still reported by more than half the surveys. Considering all macro and micronutrients investigated, no country met more than 39% WHO RNIs in all age/gender groups.

Of the 21 countries for which nutrient intakes were extracted, seven reported intakes by SEG in addition to age and gender (Estonia, Finland, France, Ireland, The Netherlands, Norway, UK). Whilst this comprises a third of countries listed in Table 3, only 13% of the 53 countries in the WHO remit represented nutrient intakes by SEG.

### 3.2. Energy and Nutrient Intakes

#### 3.2.1. Energy

**Figure 1:**
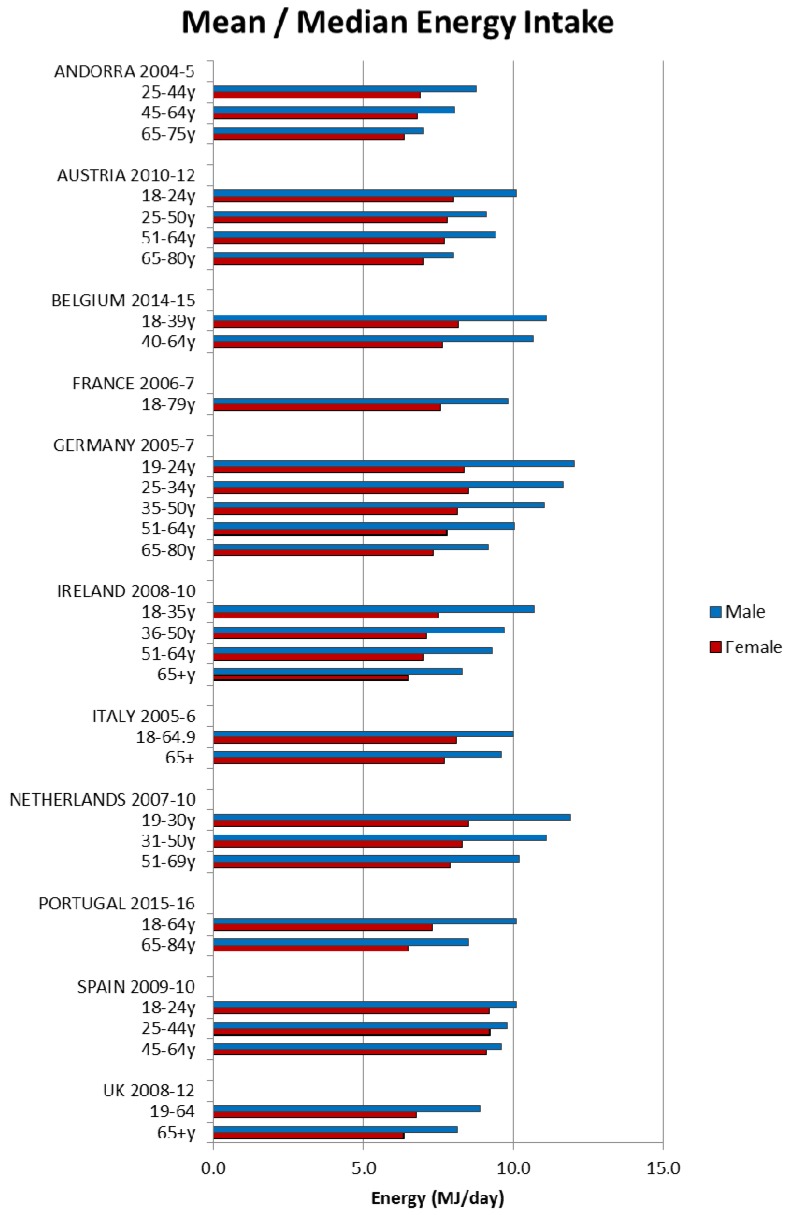
Mean/median* adult energy intake (MJ/day) for Western European countries (excluding supplements). * Figures for Spain are based on median rather than mean values.

**Figure 2:**
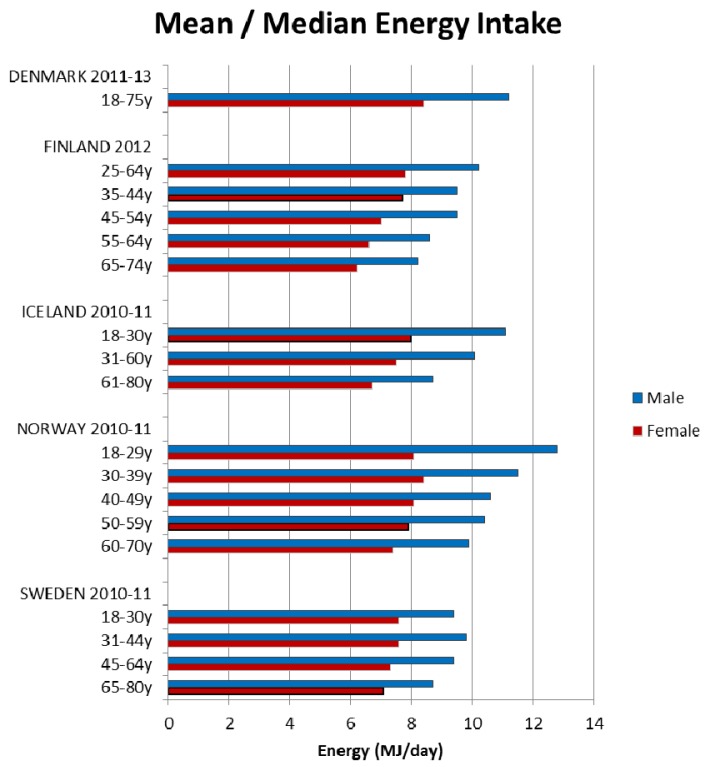
Mean/median adult energy intake (MJ/day) for Northern European countries (excluding supplements).

**Figure 3:**
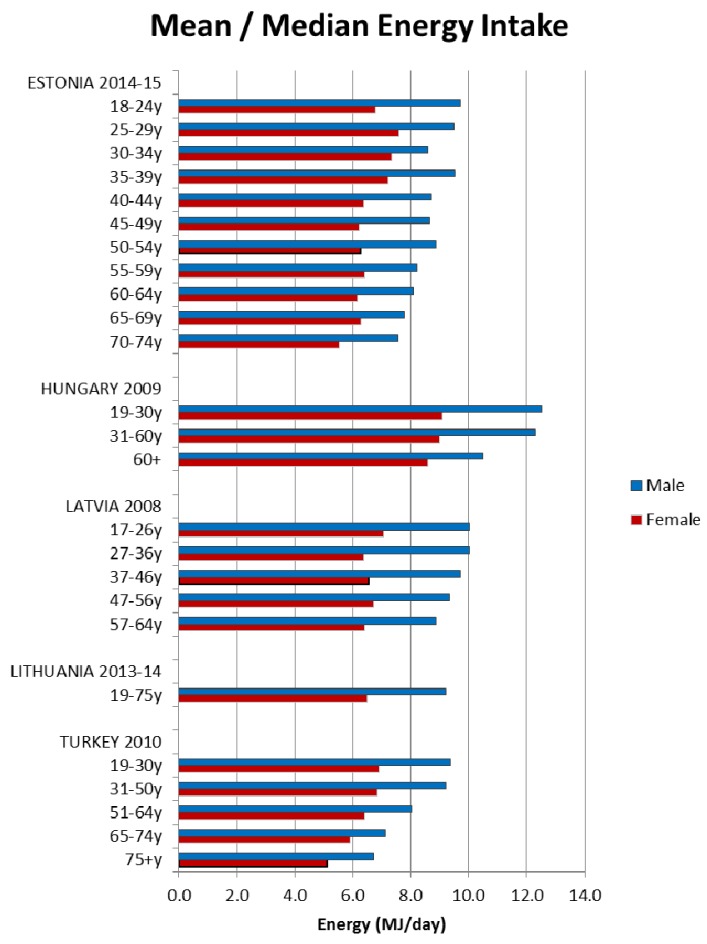
Mean/median adult energy intake (MJ/day) for Central & Eastern European countries (excluding supplements).

Energy intakes reported from the NDS have previously been documented [5]. Briefly, daily mean/median energy intakes were higher in adult males and decreased with age for all age groups in all 21 countries; however, age groupings reported were not consistent across countries (see Figure 1, Figure 2 and Figure 3).

#### 3.2.2. Macronutrients

For all macronutrients, with the exception of sugars and fibre in older age groups, males tended to have a higher intake than females in all countries across all age groups. In this section means reported are estimated weighted European means (see Table 4 and Table 5 for total weighted means by nutrient and broken down by country) and those in brackets are the ranges of gender and age group means provided in the country reports.

**Figure 4:**
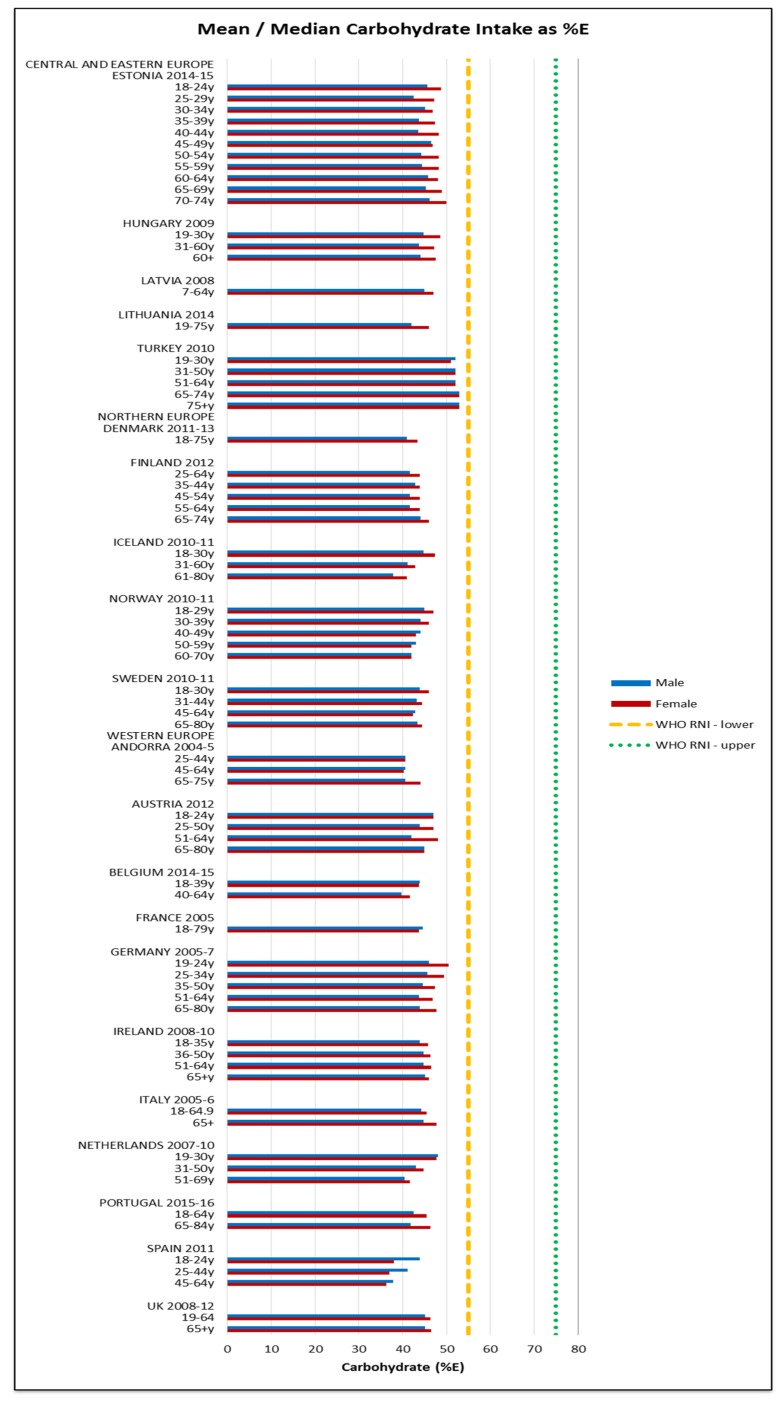
Mean/median* adult carbohydrate intake (g/day) (excluding supplements). * Figures for Spain are based on median rather than mean values.

**Figure 5:**
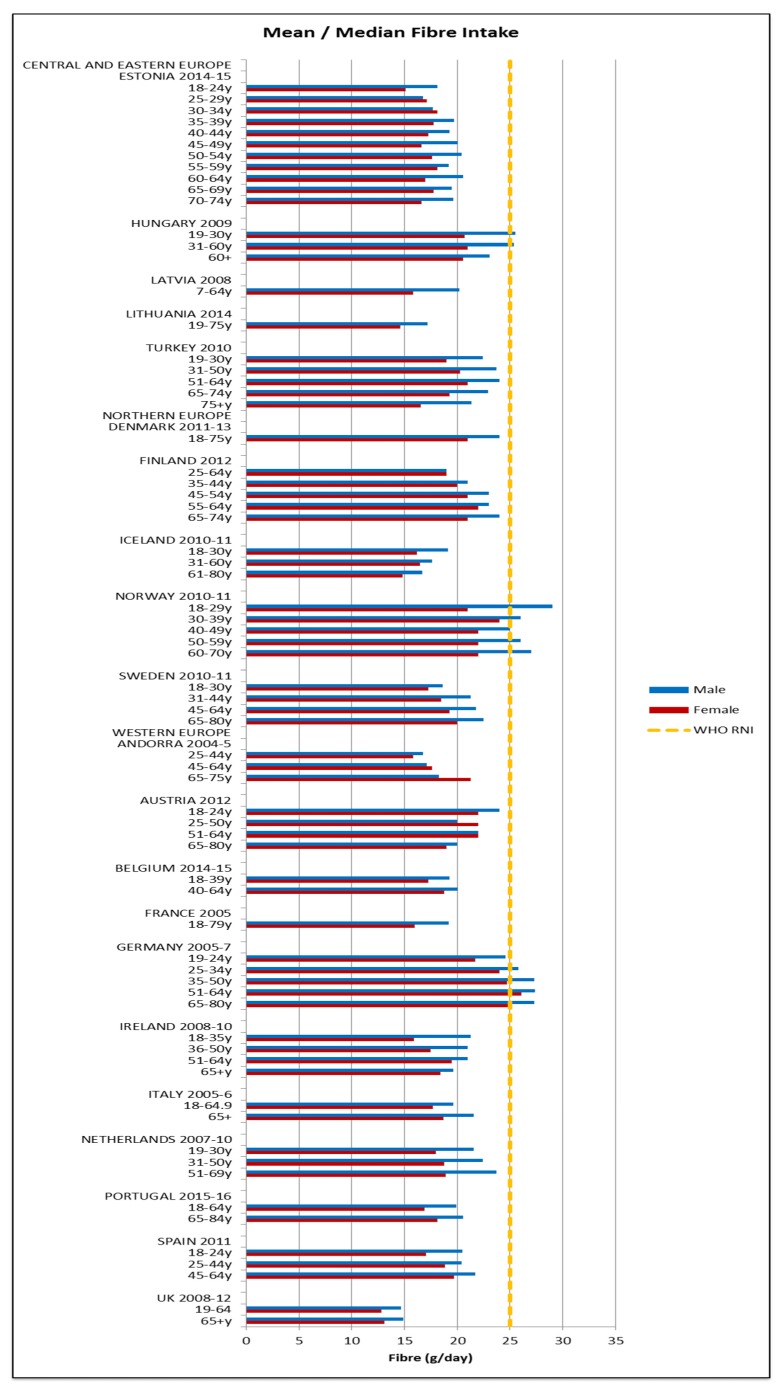
Mean/median* adult fibre intake (g/day) (excluding supplements). * Figures for Spain are based on median rather than mean values.

**Figure 6:**
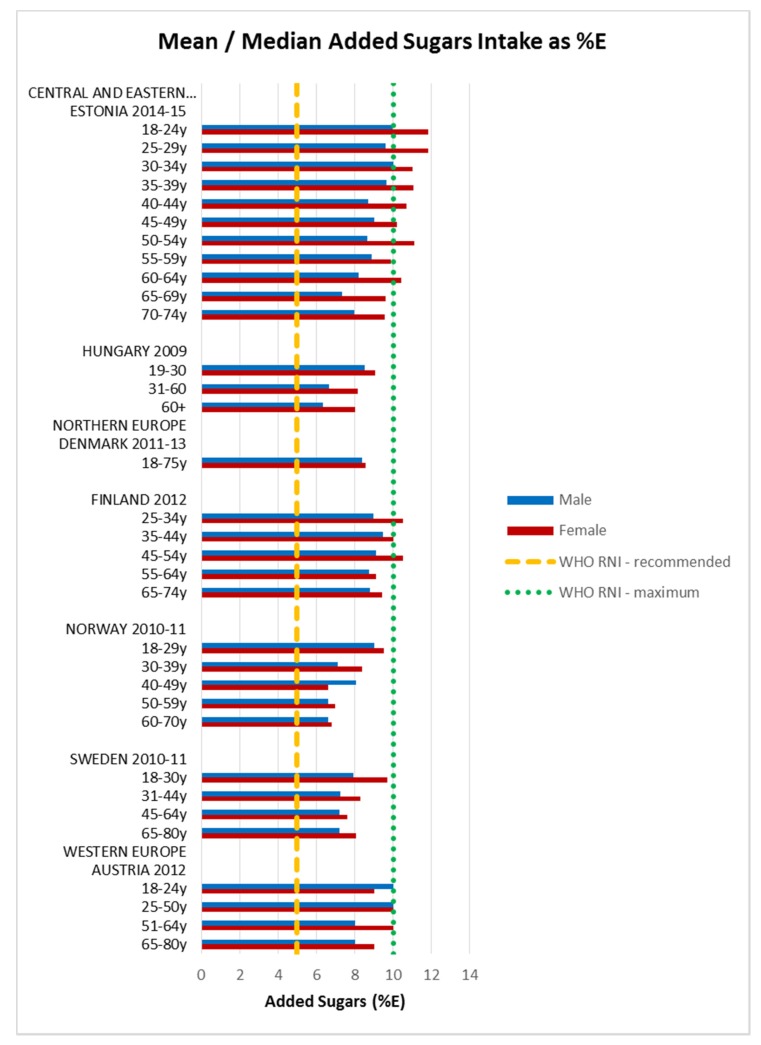
Mean/median adult added sugars intake (g/day) (excluding supplements).

Attainment of the WHO macronutrient RNIs [10] was generally poor across all regions and marginally worse in CEEC. All age groups in all countries were comfortably over the lower 10%E protein RNI in men and women. Just over half of countries met or exceeded the upper RNI of 15%E, though there was no regional pattern. No country met the lower carbohydrate RNI of 55%E in any age group (Figure 4). The mean carbohydrate intake was 209 g, (range 156–265 g) for women and 264 g (range 173–342 g) for men. Most countries fell short of the fibre RNI in all ages; only Norway (all ages), Germany (women aged 51–64 and men across the lifespan) and Hungary (non-elderly men) met the 25 g target (Figure 5). Mean fibre intakes were 19 g (range 13–26 g) for women and 21 g (range 15–29 g) in men. All countries that reported added sugars (*n* = 7) were over the 5% recommended RNI, although only Estonian and Finnish women were above the 10% maximum (Figure 6). Mean added sugar intakes were 41 g (range 30–49 g) for women and 48 g (38–69 g) in men.

**Figure 7:**
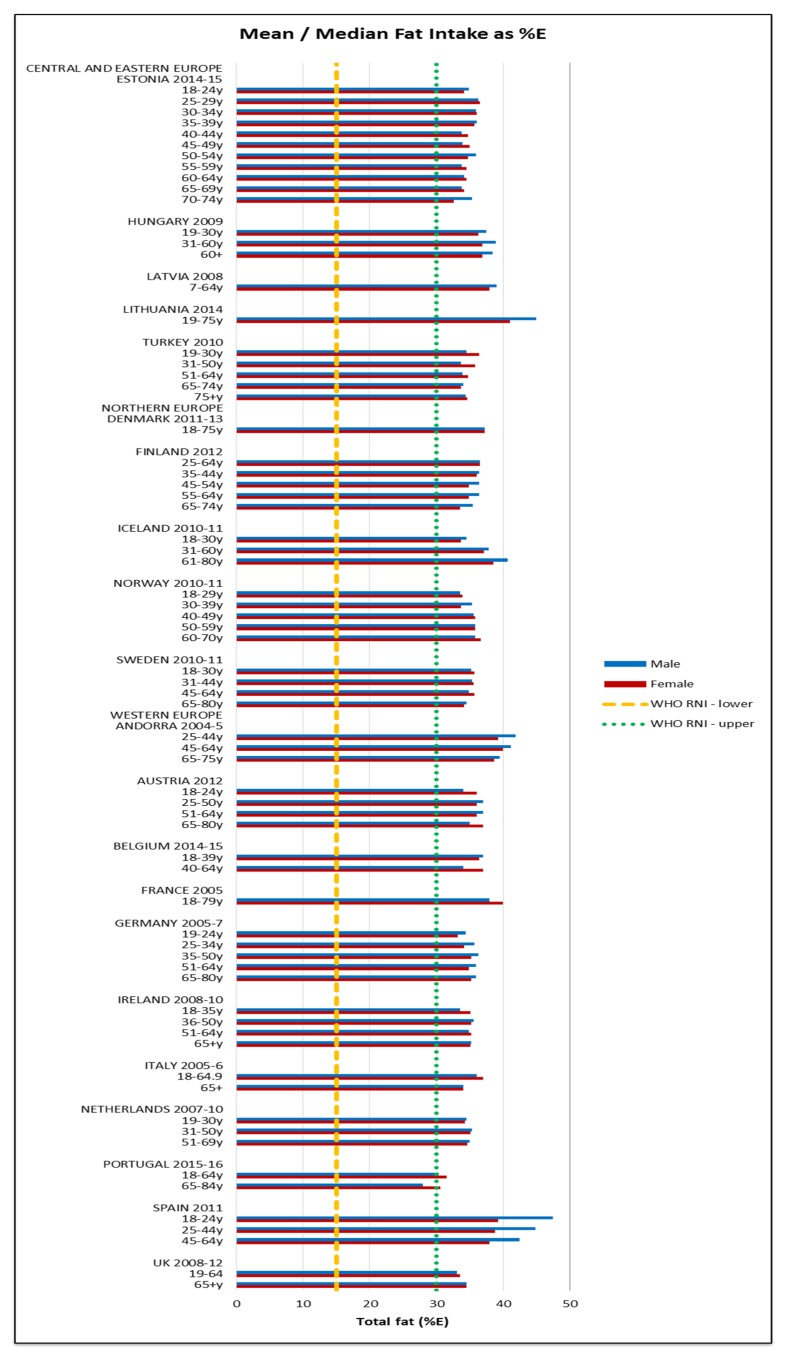
Mean/median* adult fat intake (g/day) (excluding supplements). * Figures for Spain are based on median rather than mean values.

**Figure 8:**
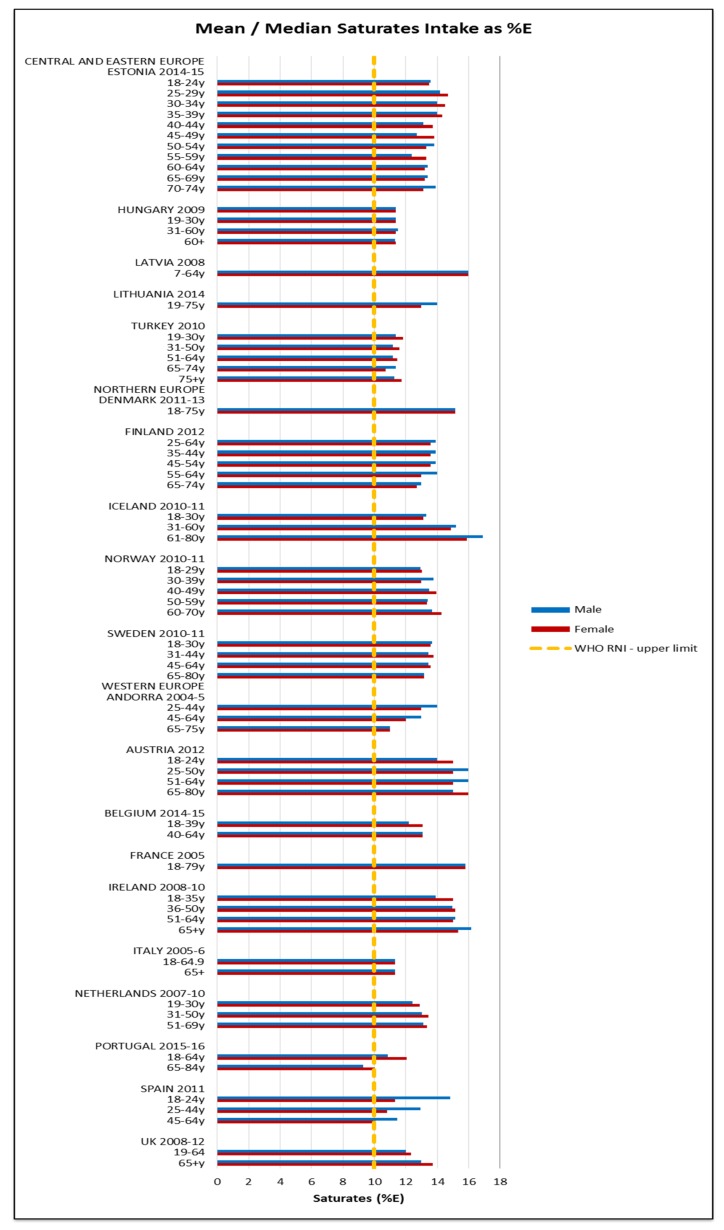
Mean/median* adult saturates intake (g/day) (excluding supplements). * Figures for Spain are based on median rather than mean values.

All countries exceeded the WHO upper fat limit of 30%E except Portuguese elderly men (Figure 7). The mean total fat intake was 73 g (51–95 g) in women and 94 g (61–127 g) in men. The majority of countries were also above the 10%E RNI for saturates; only Portuguese elderly men were below (Figure 8). The mean saturates intake was 25 g (16–33 g) for women and 32 g (20–45 g) for men. Only Lithuanian men exceeded the upper PUFA RNI of 10%E and just under half the countries were below the lower RNI of 6%E, leaving around half of countries with optimum intakes between the two RNIs; there was no regional pattern. The greatest WHO RNI compliance was in TFAs, where only Icelandic elderly men exceeded the <1%E limit with intakes at 1%E. However, only nine countries reported TFAs; the CEEC region had fewest countries reporting intakes.

Omega fats RNI attainment was mixed; 60% of countries that reported n-3 intakes were between the 1%–2%E RNI bands, mostly in Northern Europe, whilst 4 countries did not meet the lower RNI. Turkey and Hungary exceeded the upper n-6 limit of 8%E but fewer countries achieved intakes within the lower and upper RNI bands in the majority of age/gender groups than for n-3. There was no age or gender pattern but Northern European countries had higher n-3 and lower n-6 intakes.

#### 3.2.3. Micronutrients

Micronutrient RNI [11,12,13] attainment was slightly better than macronutrient, though the variation in male/female intake patterns was higher and there were no clear age patterns.

**Figure 9:**
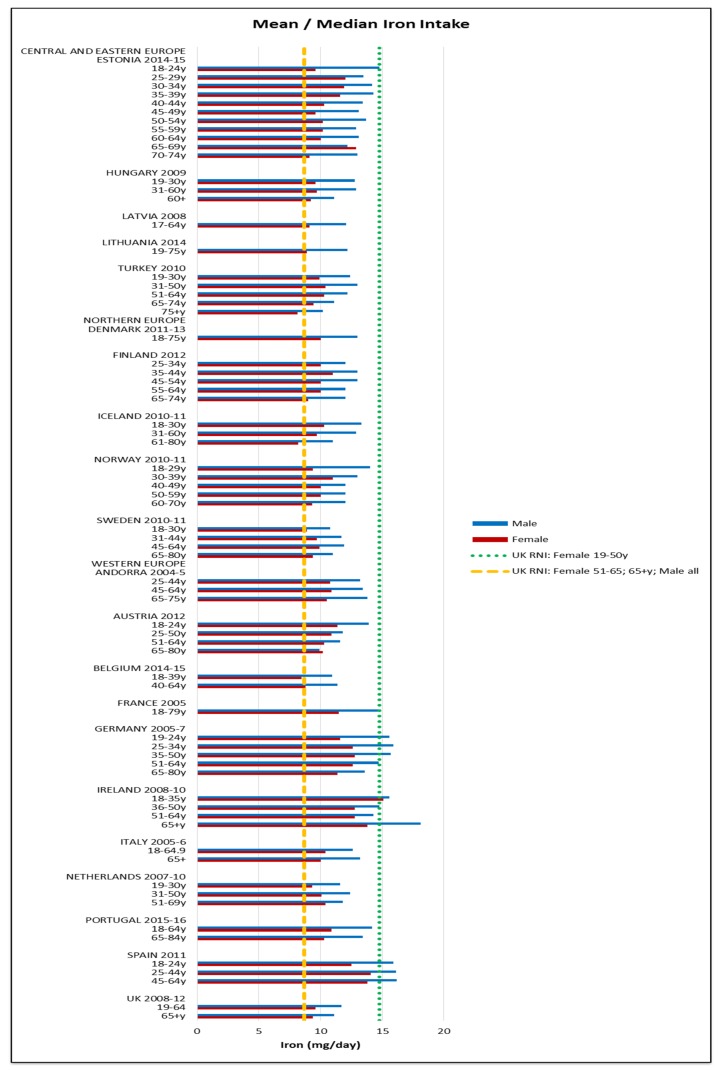
Mean/median* adult iron intake (mg/day) (excluding supplements). * Figures for Spain are based on median rather than mean values.

**Figure 10:**
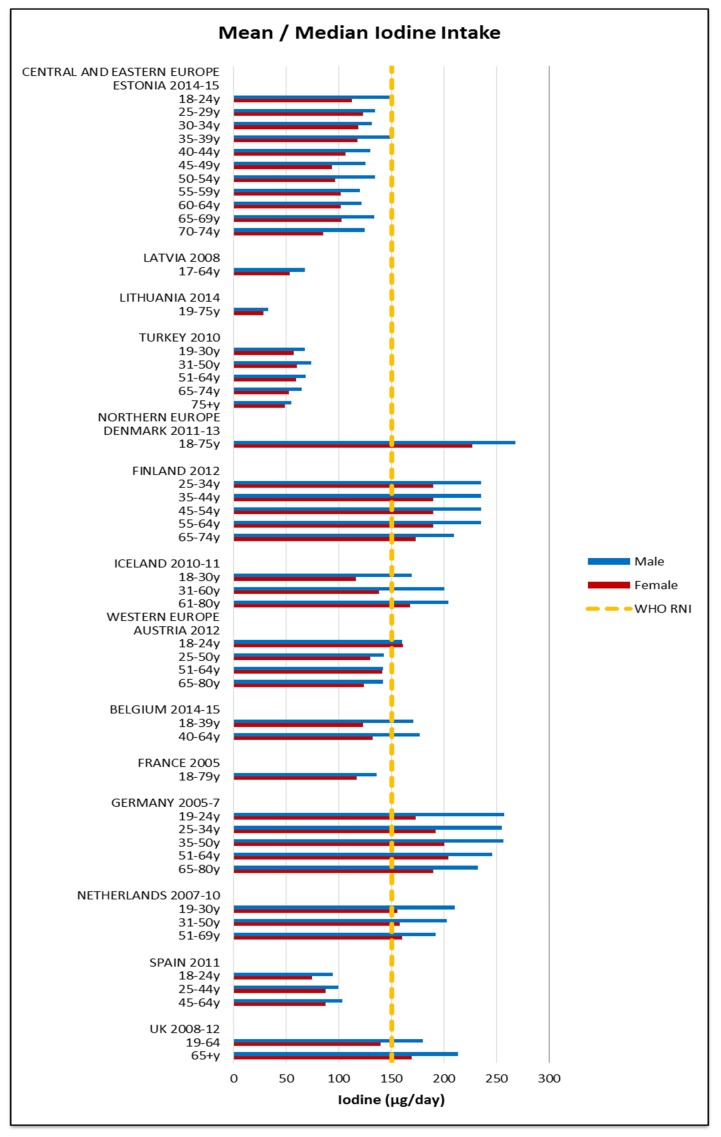
Mean/median* adult iodine intake (μg/day) (excluding supplements). * Figures for Spain are based on median rather than mean values.

**Figure 11:**
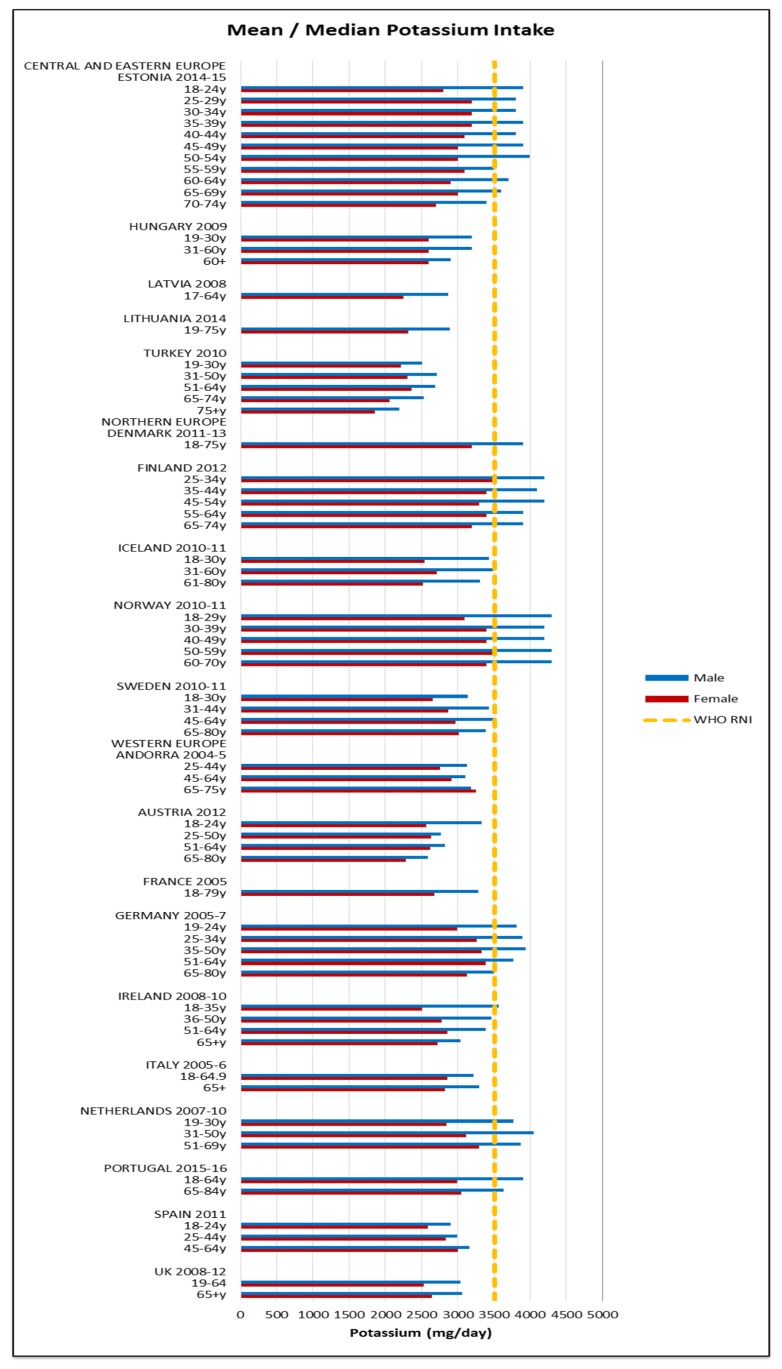
Mean/median* adult potassium intake (mg/day) (excluding supplements). * Figures for Spain are based on median rather than mean values.

All countries comfortably met the 4.9 mg female and 7 mg male RNI for zinc. The majority of countries met the 2.4 μg RNI for vitamin B12; only Lithuanian and Turkish older and elderly women and elderly men fell short. Fulfilment of iron, iodine and potassium RNIs was mixed and women generally had poorer attainment—particularly younger women (Figure 9, Figure 10 and Figure 11 respectively). For iron, only younger Irish women met the 14.8 mg UK RNI [15] for women aged 19–50, though all countries met the 8.7 mg RNI for women aged 51–65 y and 65+ y except elderly Turkish women. All countries met the 8.7 mg male RNI for iron. Mean intakes were 10.9 mg (8.1–15.1 mg) in women and 13.4 mg (9.9–18.1 mg) in men.

Just under half of countries that reported iodine met the 150 μg RNI; more men and younger age groups exceeded the RNI but there were no regional patterns. The mean iodine intake was 127 μg (28–227 μg) in women and 156 μg (33–268 μg) in men. No countries met the 3510 mg RNI for potassium in women; half of countries met the RNI in at least some male age groups, though there was no regional pattern between countries. Mean intakes were 2771 mg (1855–3500 mg) in women and 3245 mg (2192–4300 mg) in men.

**Figure 12:**
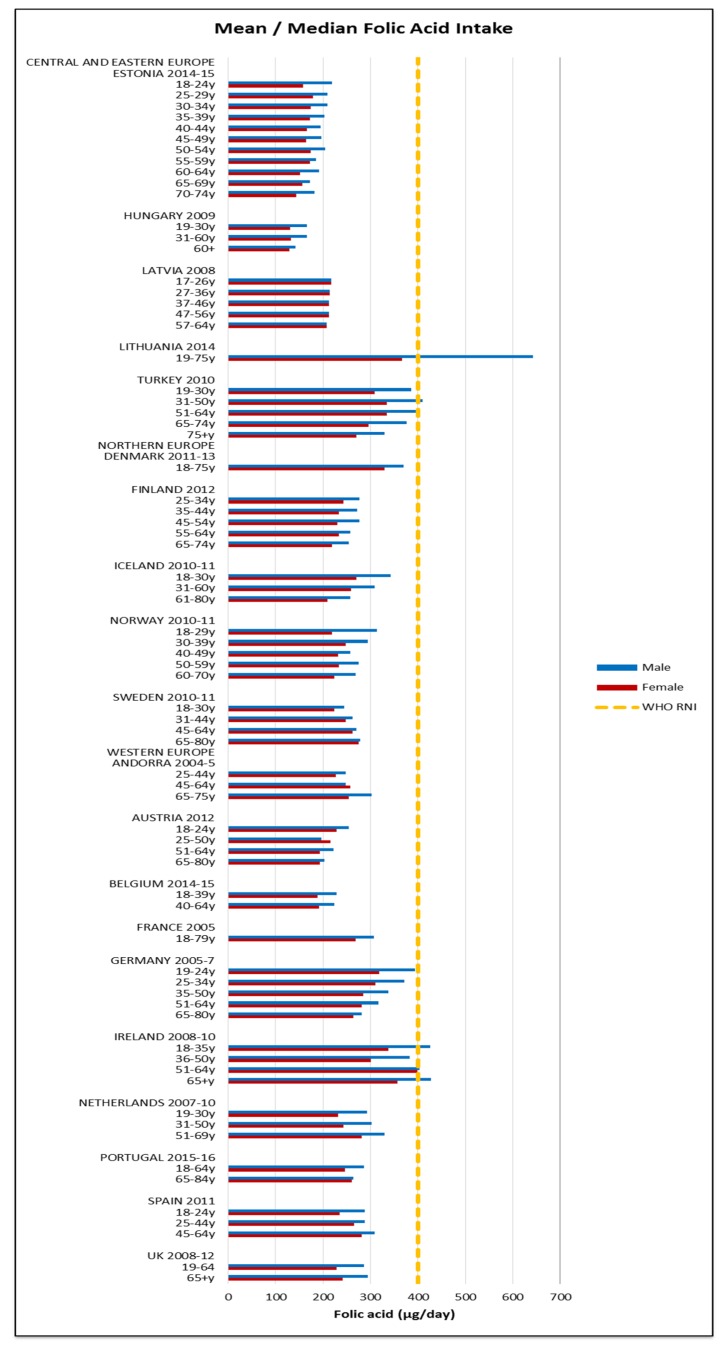
Mean/median* adult folic acid intake (μg/day) (excluding supplements). * Figures for Spain are based on median rather than mean values.

**Figure 13:**
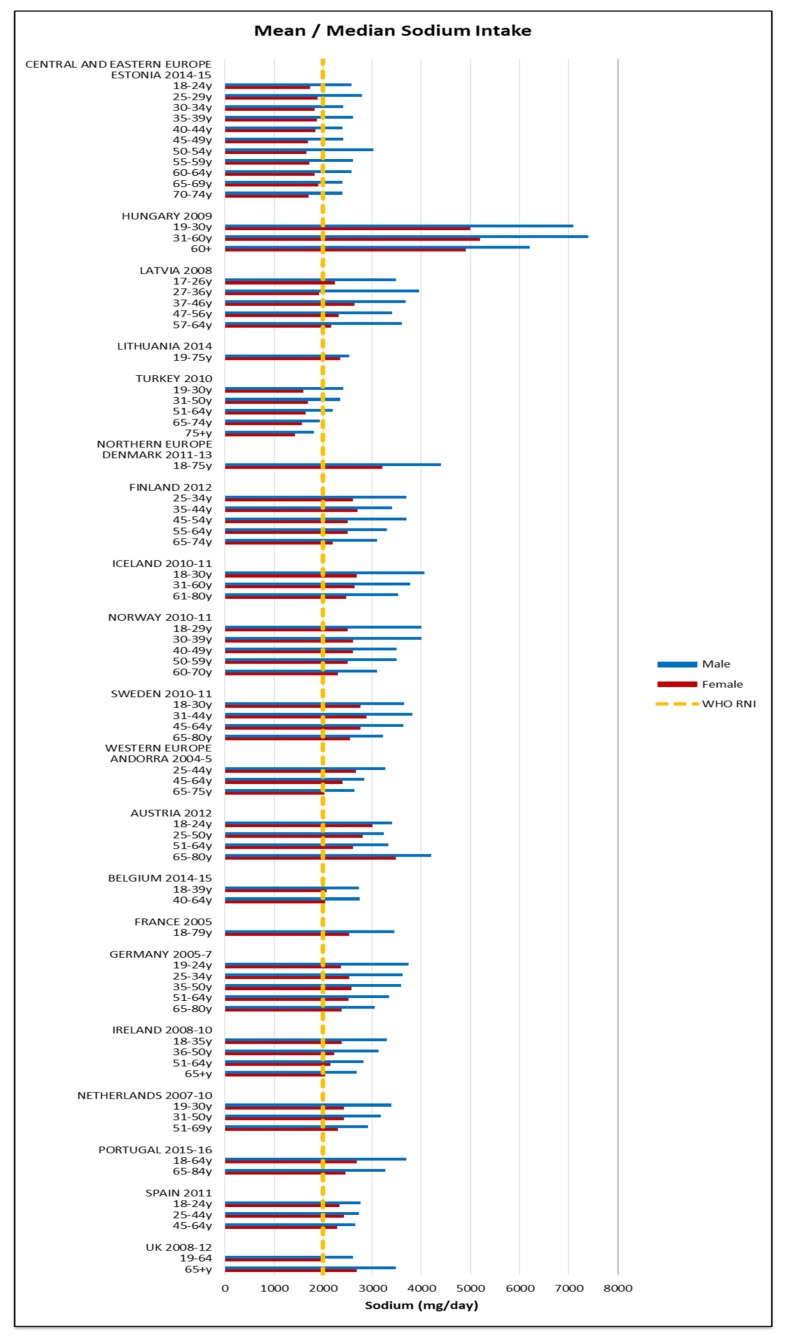
Mean/median* adult sodium intake (mg/day) (excluding supplements). * Figures for Spain are based on median rather than mean values.

Few countries and no women of any nationality met the 400 μg RNI for folic acid; only young and elderly Irish men and middle-aged Lithuanian and Turkish men had adequate intakes (Figure 12). The mean folic acid intake was 268 μg (129–399 μg) in women and 318 μg (142–643 μg) in men. The majority of countries over-consumed sodium; all male age groups exceeded the 3000 mg RNI and in women only the UK and younger Estonian and Latvian women did not (Figure 13). Mean sodium intakes were 2341 mg (1426–5200 mg) in women and 3163 mg (1811–7400 mg) in men.

**Figure 14:**
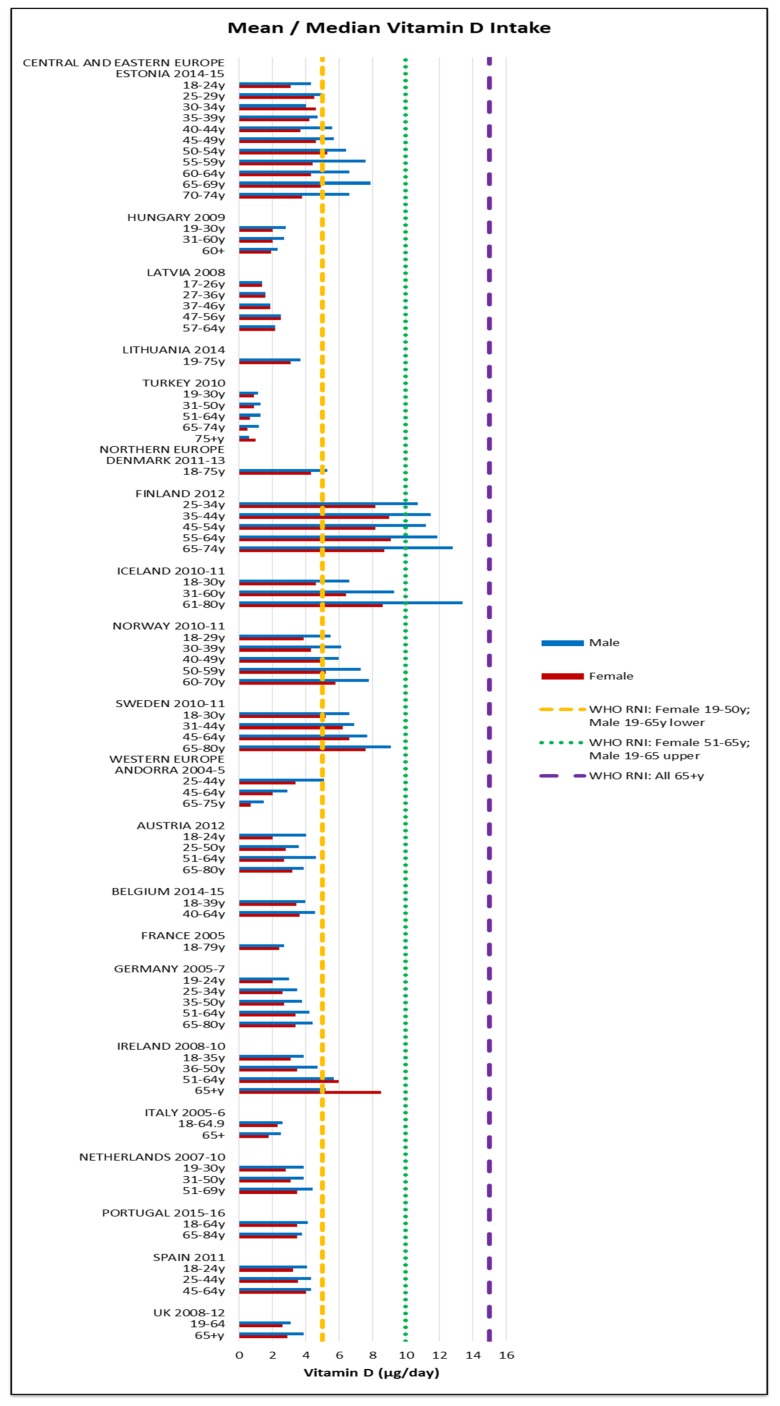
Mean/median* adult vitamin D intake (μg/day) (excluding supplements). * Figures for Spain are based on median rather than mean values.

**Figure 15:**
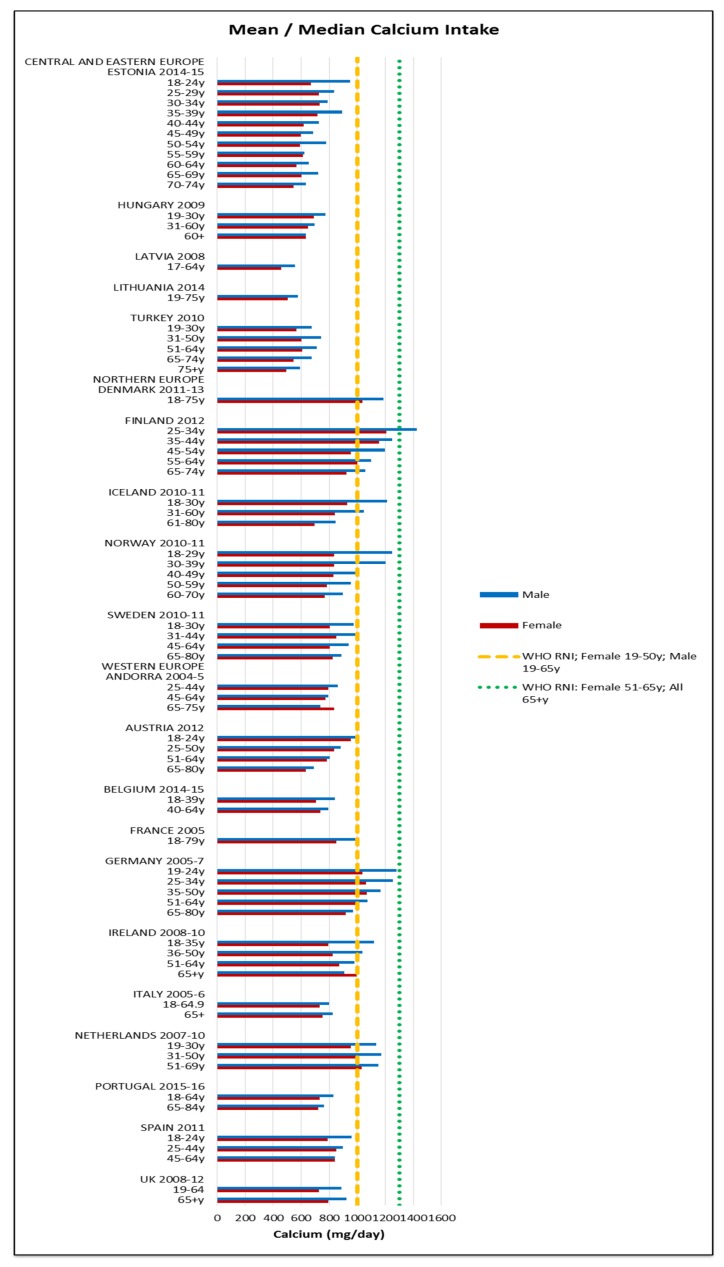
Mean/median* adult calcium intake (mg/day) (excluding supplements). * Figures for Spain are based on median rather than mean values.

Assessing RNI attainment in vitamin D and calcium (Figure 14 and Figure 15) is made more difficult by different ages having different RNIs—where age groupings span RNI categories it cannot be specified whether or not the RNI is met. Where this could be assessed, few countries met the RNI for the age range in question, particularly in women and the elderly, where no countries met the RNI. Mean vitamin D intakes were 2.7 μg (0.5–9.1 μg) in women and 3.3 μg (0.6–13.4 μg) in men. Mean calcium intakes were 799 mg (457–1206 mg) for women and 908 mg (555–1424 mg) in men.

## 4. Discussion

### 4.1. Data Extracted

This review details the provision of energy and nutrient intake data in nationally representative surveys across the 53 countries of the WHO Europe region for nutrients of particular concern to the WHO European Region [1]. Only 40% (*n* = 21) of countries provided intake data by gender and age group for adults; the majority of these were Western and Northern European countries. This implies that nutrition policies in the remaining 60% of countries without intake data may be based on limited evidence, particularly in CEEC. This is a concern, as overweight and obesity have tripled in some of these countries since 1980 and NCD prevalence rates are reaching those of Western Europe [1]. Additionally, unknown pockets of micronutrient deficiencies may exist in some countries. 

Although the surveys used different dietary methodologies, we felt it important to report intakes in their publicly available format. Of the 21 surveys for which intakes were extracted, energy, macro and micronutrients were generally well represented and there were no apparent regional patterns in nutrient intake gaps. This provides a good basis for assessing population status and identifying vulnerable gender/age groups in these countries (see Appendices A & B). The biggest gaps in macronutrient provision were TFA, omega fatty acids and sugar, the latter particularly in CEEC, which have been identified as nutrients of concern [1,49]. These are therefore important knowledge gaps, as without intake data, population and subgroup status cannot be known or appropriate policies devised. Iodine was reported by the least surveys; deficiencies remain frequent in WHO Europe [1] and even mild-moderate maternal deficiency is associated with decreased cognitive function in children [50]. This gap therefore limits effective policy formation to address population groups most in need.

A third of countries, or just 13% of the 53 WHO Europe countries, reported energy and nutrient intakes by SEG (Table 3). This is concerning, as whilst NDS could be used to identify subgroups lacking nutrients based on gender and age, few can gauge the potential for NDS to capture socio-economic inequalities. In addition, different, often multiple variables were used to represent SEG, making inter-country comparisons difficult. Consequently, vulnerable groups across Europe may be at risk of malnutrition through under or over-nutrition and related NCDs, with limited means for governments and health bodies to measure, monitor or address in policy.

### 4.2. Energy Intakes

Energy intakes did not vary substantially by European region, although the different dietary assessment methodologies employed by surveys may make inter-country comparisons unreliable. In addition, under-reporting is associated with all dietary assessment methods, including the 24 h recall and food diaries used by the surveys in question [51], which could impact on the validity of intake data and the conclusions derived from it. Most surveys either included under-reporters or did not specify—only Belgium explicitly excluded under-reporters, which may elevate Belgian intakes compared to the other countries. 

### 4.3. Nutrient Intakes and WHO RNI Status

WHO RNI attainment was low across all regions—only Finland and The Netherlands met more than a third of WHO RNIs in all gender/age groups, suggesting that nutritional concerns exist across WHO Europe and that population groups within countries are not impacted equally. Turkey had the lowest intakes in most nutrients, potentially because it reported the oldest age grouping (75+ y) who may be likely to consume less than younger adults. However, the Turkish 65–74 y group also had low intakes for multiple nutrients compared to equivalent age groups in other countries.

### 4.4. Carbohydrates and Fats

The majority of countries did not meet the carbohydrate, sugar or fiber guidelines. This suggests a potential under-consumption of complex carbohydrates, going against established dietary advice [10], particularly The Netherlands, which had a lower fiber but high sugar intake.

Most countries exceeded fat and saturates guidelines. Andorra and Lithuania had modest absolute but high %E intakes, suggesting a diet with an unfavorable fatty acid composition, particularly in Andorra, which does not have the high %E in PUFA evident in Lithuania. This could lead to increased susceptibility to NCDs like coronary heart disease (CHD) [52]. Similarly, Denmark, Norway and Iceland had a high saturates intakes without correspondingly high unsaturated fat intakes. This suggests that Northern European countries may have higher saturated fat intakes as a proportion of total fat, possibly reflecting unfavorable national dietary patterns, though diet is one of many contributors to disease susceptibility.

Spain, Italy and Andorra had high MUFA intakes, which could indicate a Mediterranean diet pattern, linked to reduced all-cause mortality and NCD risk [53,54]. Hungary, Lithuania and Turkey had high PUFA intakes, which could indicate a regional influence based on CEEC diet patterns, particularly in Turkey, which had low intakes for most macronutrients other than PUFA. This pattern is also evident in n-6 intakes—both Turkey and Hungary exceeded the upper WHO RNI. TFAs had the greatest RNI compliance, possibly due to a combination of health bodies like WHO calling for a wholesale TFA reduction [1] and widespread TFA-reduction policies across Europe, including bans, labelling initiatives and voluntary product reformulation [55,56,57,58].

Of those reporting omega fats, Northern European countries had higher n-3 but lower n-6 intakes. This could potentially be a function of national diet patterns such as high oily fish consumption in Scandinavia; of the five European countries participating in the European Food Consumption Validation Project (EFCOVAL), Norway had the highest fish consumption [59]. Although some countries reported different n-3 and n-6 variants, the highest intakes were not necessarily those that included multiple variants. Therefore, although amalgamated n-3 and n-6 levels may not represent the full population omega intake, this does not necessarily invalidate inferences made. It does, however, highlight the need for a common methodological approach to conducting dietary surveys and gathering nutrient intake data.

### 4.5. Micronutrients

The percentage of CEEC that surveyed micronutrients generally had lower micronutrient intakes than the other regions, particularly Lithuania and Turkey—exceptions were relatively high Lithuanian folate and Hungarian, particularly male, sodium intakes. This suggests the potential for population groups to have suboptimum diets with excessive or inadequate intakes of particular nutrients. More research is necessary to determine whether this is a function of typical regional diet patterns and to inform debate on potential solutions such as food-based compared to fortification and/or supplementation for specific at-risk groups.

The majority of countries not meeting the iodine RNI were CEEC (Figure 10); this could be attributed to regional differences in salt iodization practices. However, patterns are difficult to elucidate, as salt-iodization programs are not uniform within or between countries and even where countries have policies, household coverage may be low [60]. For sodium only the UK and CEEC females did not exceed the RNI, although sodium intakes from dietary records may be unreliable. This could reflect generally low CEEC intakes and also the UK being an early adopter of a comprehensive voluntary salt reduction program since 2008 [61,62], which is credited with facilitating a reduction in salt intakes [63]. However, care must be taken when considering salt reduction, as salt iodization is a primary means of improving iodine intakes [64]. European iodine status is concerning; of the WHO regions Europe has the highest deficiency level. Potential solutions for compatibility, such as increasing the concentration of iodine in salt or using alternative vehicles, may need to be considered in countries where iodine status is poor.

Nordic countries had higher mineral intakes, whilst different national fortification practices may explain some variations in vitamin intake. Scandinavian vitamin D intakes were relatively high, with the exception of Denmark and Swedish vitamin D fortification is more extensive than Danish [65]. Northern European countries have less sunlight, meaning populations are likely to need more vitamin D from food, so where fortification is low, intakes are likely to be lower. This review includes fortification in base diet, as most countries’ food composition databases do not separate this out [66].

Our findings support the identification of iodine, iron and vitamin D by WHO as nutrients of concern [1], particularly in CEEC, women and the female elderly respectively. Women and the female elderly appear to be the most vulnerable groups across the countries, with additional risk of potassium, calcium and folate deficiency. The latter is of particular concern in women of reproduction age as it can prevent neural tube defects in offspring [67]. Nutrients of universal concern were carbohydrates, fats and sodium. In addition to improving micronutrient intakes, increasing complex carbohydrate and fiber consumption and reduction of sodium, fat and saturates should be a priority across the majority of European population groups.

### 4.6. Strengths and Limitations

The strengths of this review are that it provides a unique, current account of reported energy and nutrient intakes for adults across whole populations and subgroups in Europe, with reference to WHO RNI attainment. The review will help identify where there is a need to improve diets and could enable governments and health bodies to better use NDS to reduce NCDs and related conditions across Europe. It also details where surveys report nutrient intakes by SEG—future work could present and assess intakes by SEG in more detail.

A limitation is that inconsistent age groupings across countries made inter-country comparisons difficult. In Andorra, the youngest age group spanned both adults and children, invalidating conclusions regarding adults aged 18–24. Further investigations using raw data could obtain more reliable conclusions via consistent age groups. Differences in dietary assessment methodologies present further limiting factors when making inter-country comparisons. For example, mean energy intakes in young Norwegian men were 3.4MJ higher than in the same age group in Sweden, despite being neighboring countries whose NDS were conducted in the same years. These differences could therefore be either due to the different methodological approaches employed, or a genuine intake disparity. In addition, collection over more days better reflects usual intake due to greater control over day-to-day variation [68]. However, most countries did not employ usual intake procedures such as the Statistical Programme to Assess Dietary Exposure (SPADE) [68]. This could affect intakes, although the impact would be greater on the distribution rather than the mean values. Some countries did not report overall country means for nutrients by gender, so a consistent weighting method was used for all countries. However, the overall country means we tabled are approximations based on the assumption that the numbers in each age group are proportionate to those in the total population. Due to availability, we used total national population numbers, which included adults and children, to calculate weighted regional and overall European means; therefore, means of countries with larger proportions of children in their populations may be given more weighting than required in these approximations.

Lack of alignment and completeness of national food composition databases and classification systems represents a further limitation. Sweden used sucrose as a proxy for added sugar [45], whilst others did not specify, so the number of mono and disaccharides included may differ and intake levels be incomparable. In this review, sucrose was equated with added sugars. If differences like these exist in other countries, estimated intake levels may vary as a result. Different composition databases may represent nutrients to different degrees; of the 14 countries reporting iodine, for example, not all may have iodine values for all foods. Consequently, intake values for particular nutrient in certain countries may be less accurate. In addition, the nutrient values underpinning food composition databases may be derived from different analytical methods, as with folate, preventing true data harmonization and potentially skewing intakes. This could explain the particularly low UK fiber intakes; the UK survey used the Englyst method, whereas other countries may have used AOAC or other methods. Whilst there is good agreement between methods in most foods, the Englyst method produces lower results in certain cereals, fruits, white beans and peanuts, which may affect fiber intake levels [69]. Additionally, food composition databases may not accurately reflect fortification—not all countries’ food composition databases account for iodine fortification, potentially depressing intake estimates [70]. Some food composition databases may not be updated to account for reformulated products; for instance, TFA values reported may be higher than those found in purchased products [71].

Future research could investigate how methodological differences impact on intake estimates in European populations—low Turkish intakes may have been due to either socioeconomic or methodological factors, using only a single 24 h recall [70]. Ireland had high vitamin intakes and was the only country that used weighed intake; the majority of countries used 24 h recall [5], which Holmes & Nelson [72] rank as less likely than weighed intakes to generate accurate portion size data.

## 5. Conclusions

This review has found that adult energy and nutrient intakes could only be extracted from 21 (40%) of the 53 WHO Europe countries and that methodological and other differences make inter-country comparisons difficult. The main gaps lie in CEEC, where nutrition policies may therefore be based on limited evidence, with a lack of data meaning potential unknown nutrient deficiencies may exist. Macro and micronutrients of interest were reported by most countries with intake data, though TFAs, omega fats, sugars and iodine had the least coverage. WHO RNI attainment was generally poor, particularly for macronutrients and was most notably lacking in women. Concerning micronutrients, the same was seen and was most prominent amongst the elderly female population and CEEC. Only 13% of WHO Europe countries reported intakes by SEG and by different methods. Consequently, the majority of WHO Europe countries are unable to adequately assess and address nutrient deficiencies in vulnerable SEGs. Future efforts should encourage WHO Europe countries to report a full range of nutrient intakes, including by SEG, in a uniform way.

## Figures and Tables

**Table 1:** Survey inclusion and exclusion criteria.

Included	Excluded
Surveys conducted at an individual level	Surveys collected at group i.e. household level
Nationally representative surveys	Non-nationally representative, regional only surveys
Results of surveys reported by published and unpublished reports, academic journals and websites	Surveys with data collected prior to 1990
Surveys that included individuals >2 y	Surveys with samples exclusively <2 y
Surveys based on whole diet rather than specific food groups	Surveys with incomplete food group coverage
	Surveys with small sample sizes (*n* < 200)

**Table 2:** Nutrients of interest in dietary surveys.

Macronutrients	RNI	Micronutrients	RNI
Energy (MJ and kcal)	N/A	Folic acid (μg)	Minimum
Carbohydrates (g and %Energy (E))	Target	Vitamin B12 (μg)	Minimum
Sugars (g)	Maximum	Vitamin D (μg)	Target
Sucrose (g)	Maximum	Calcium (mg)	Minimum
Starches (g)	N/A	Potassium (mg)	Minimum
Fiber (g)	Target	Sodium (mg)	Maximum
Total fat (g)	Maximum	Iron (mg)	Minimum
Saturates (g)	Maximum	Iodine (μg)	Minimum
Monounsaturated fatty acids (MUFA) (g)	N/A	Zinc (mg)	Minimum
Polyunsaturated fatty acids (PUFA) (g)	Target		
Trans Fatty Acids (TFAs) (g)	Maximum		
Protein (g)	Target		
Omega fatty acids (g)	Target		

**Table 3:** National diet surveys across countries in WHO Europe 1990–2016 with nutrient intakes reported.

Country	Survey Name	Survey Year	Source *	Sample Size	Sample Age	Dietary Methodology	Nutrient Reference Database	Nutrient Intakes by SEG Y/N **	WHO RNIs Not Met by All Age Groups (%) ^Ϯ^	Reference
Andorra	Evaluation of the Nutritional Status of the Andorran Population	2004–2005	4	900	12–75	24 h recall (×2 for 35% sample), FFQ	CESNID. *Tablas de composición de alimentos*. Barcelona: Edicions Universitat de Barcelona-Centre d’Ensenyament Superior de Nutrició i Dietètica, 2002	N	83	[17]
Austria	Austrian nutrition report 2012 (OSES)	2010–2012	2	1002	7–14; 18–80	3-day diary (consecutive) (children); 2*24 h recall (adults).	Analysis run with software “(nut.s) science” based on Bundeslebensmittelschlüssel 3.01/Goldberg cut-offs for data cleaning	N	72	[18]
Belgium	Belgium National Food Consumption Survey (BNFCS) 2014	2014–2015	1/2	3146	3–64	2*24 h recall	The NIMS Belgian Table of Food Composition (Nubel); Dutch NEVO	N	78	[19,20]
Denmark	Danish National Survey of Diet and Physical Activity (DANSDA) 2011–2013	2011–2013	2	3946	4–75	7-day diary (consecutive)	Danish Food Composition Databank	N	67	[21]
Estonia	National Dietary Survey	2014–2015	1	4906	4 m–74 y	2*24 h recall (age > 10); 2*24 h food diary (age < 10); FFQ (age > 2)		Y—income, poverty threshold, education	78	
Finland	The National FINDIET 2012 survey (FINRISK)	2012	2	1708	25–74	48 h recall	Fineli 7 Food Composition Database	Y—education	61	[22]
France	Individual National Food Consumption Survey (INCA2)	2006–2007	2	4079	3–79	7-day diary (consecutive)	Food Composition Database of CIQUAL of Afssa	Y—education	83	[23]
Germany	German National Nutrition Survey (Nationale Verzehrstudie) II (NVSII)	2005–2007	1/3	15,371	14–80	DISHES diet history interview, 24 h-recall, diet weighing diary (2*4 days)	Bundeslebensmittelschlüssel (BLS)	N	78	[24,25]
Hungary	Hungarian dietary survey 2009	2009	2	3077	19–30, 31–60, 60+	3-day diary, FFQ,	Új tápanyagtáblázat	N	72	[26,27]
Iceland	The Diet of Icelanders—a national dietary survey 2010–2011	2010–2011	1	1312	18–80	2*24 h recall + FFQ	Icelandic Database of Food Ingredients (ÍSGEM); Public Health Institute for Raw Materials in the Icelandic Market	N	72	[28,29]
Ireland	National adult nutrition survey 2011 (NANS)	2008–2010	1	1500	18–90	4-day semi weighed food diary (consecutive)	McCance and Widdowson’s The Composition of Foods 5&6 editions	Y—social class and education	72	[30,31]
Italy	The third Italian National food consumption survey INRAN-SCAI 2005–2006	2005–2006	2	3323	0.1–97.7	3-day diary (consecutive)	Banca Dati di Composizione degli Alimenti	N	83	[32]
Latvia	Latvian National Food Consumption Survey 2007–2009	2008	1	1949	7–64	2*24 h recall, FFQ	Latvian National Food Composition Database 2009	N	78	[33]
Lithuania	Study of actual nutrition and nutrition habits of Lithuanian adult population	2013–2014	1	2513	19–75	24 h recall + questionnaire	EuroFIR Food Classification	N	83	[34]
The Netherlands	Dutch National Food Consumption Survey 2007–2010 (DNFCS 2007–2010)	2007–2010	1/2	3819	7–69	2*24 h recalls	Dutch Food Composition Database (NEVO)	Y—education	61	[35,36,37]
Norway	Norwegian national diet survey NORKOST3	2010–2011	2	1787	18–70	2*24 h recall and FFQ	The Norwegian Food Composition Tables	Y—education	83	[38]
Portugal	National Food and Physical Activity Survey (IAN-AF)	2015–2016	4	4221	3 m–84 y	2*24 h recall (non-consecutive) and FPQ (electronic interview) 2-day food diary for children <10 y	Portuguese Food Composition Table (INSA)	N	78	[39,40]
Spain	ENIDE study (Sobre datos de la Encuesta Nacionalde Ingesta Dietética)	2009–2010	2	3000	18–24; 25–44; 45–64	3-day diary + 24 h recall (consecutive)	Tablas de Composición de Alimentos, 15th ed	N	83	[41,42,43,44]
Sweden	Riksmaten 2010–2011 Swedish Adults Dietary Survey	2010–2011	2	1797	18–80	4-day food diary (consecutive)	NFA Food Composition Database	N	78	[45]
Turkey	Turkey nutrition and health survey 2010 (TNHS)	2010	2	14,248	0–100	24 h recall, FFQ	BEBS Nutritional Information System Software; Turkish Food Composition Database	N	78	[46,47]
UK	National Diet and Nutrition Survey Rolling Programme (NDNS RP 2008–2012)	2008–2012	2	6828	1.5–94	4-day diary (consecutive)	McCance and Widdowson’s The Composition of Foods integrated dataset	Y—income	72	[48]

* 1 = email contacts; 2 = general internet searches; 3 = Micha et al. [9]; 4 WHO Global Nutrition Policy Review 2017 extracted information. ** Countries that have reported nutrient intakes by socio-economic group (SEG) in addition to age and gender. **^Ϯ^** For those countries that do not report all nutrients, the RNIs for nutrients not reported are considered not met.

**Table 4:** Weighted means * by country for macronutrient in 21 national dietary surveys in the WHO Europe region.

COUNTRY	Energy (MJ)	Protein (g)	CHO (g)	Sugars (g)	Sucrose (g)	Starch (g)	Fibre (g)	Total Fat (g)	Saturates (g)	MUFA (g)	PUFA (g)	TFA (g)	n-3 (g)	n-6 (g)
***Estonia***	**National Dietary Survey 2014–2015**													
Female	6.7	64	194				17	65	26	24	11	0.5	1.8	8.2
Male	8.7	86	235				19	83	32	31	14	0.6	3.2	10.9
***Hungary***	**Hungarian Dietary Survey 2009**													
Female	8.9	79	253		44		21	87	26	27	22		0.9	21.6
Male	12.0	106	315		50		25	122	36	40	29		1.2	28.4
***Latvia***	**Latvian National Food Consumption Survey 2007–2009**													
Female	6.4	55	190				16	68	28	24	11			
Male	8.9	79	246				20	93	38	33	15			
***Lithuania***	**Study and evaluation of actual nutrition and nutrition habits of Lithuanian adult population 2013–2014**													
Female	6.5	56	178	56			15	71	22	27	16			
Male	9.2	75	224	55			17	108	34	41	24			
***Turkey***	**Turkey nutrition and health survey 2010 (TNHS)**													
Female	6.5	50	197				20	61	20	22	16		1.1	14.5
Male	8.6	67	260				23	78	26	28	19		1.4	17.4
**CEEC TOTAL Female**	6.7	53	202	56	44		20	64	21	23	16	0.5	1.1	15.2
**CEEC TOTAL Male**	9.0	72	264	55	50		23	84	28	30	20	0.6	1.4	18.5
***Denmark***	**Danish Dietary habits 2011–2013**													
Female	8.4	76	211		43		21	83	33	31	13	1.3		
Male	11.2	101	269		56		24	111	45	41	17	1.7		
***Finland***	**The national FINDIET 2012 survey**													
Female	7.0	70	181		42		21	67	26	24	12	0.8	2.8	8.7
Male	9.1	91	225		49		22	88	34	32	15	1.1	3.5	11.0
***Iceland***	**The Diet of Icelanders—a national dietary survey 2010–2011**													
Female	7.4	76	188	87			16	72	29	23	12	1.5	2.9	9.0
Male	10.0	106	240	104			18	99	40	32	16	2.2	3.8	11.9
***Norway***	**Norkost3 2010–2011**													
Female	8.0	81	205		36		22	75	29	25	14			
Male	10.9	112	278		48		27	102	39	34	19			
***Sweden***	**Riksmaten 2010–2011 Swedish Adult Dietary Survey**													
Female	7.4	72	193		37		19	70	27	26	12		2.5	8.6
Male	9.3	92	238		41		21	87	33	33	14		2.9	10.5
**NORTH TOTAL Female**	7.6	74	197	87	39		20	73	28	26	13	1.1	2.6	8.6
**NORTH TOTAL Male**	10.0	98	250	104	47		23	95	37	35	16	1.4	3.1	10.7
***Andorra***	**Evaluation of the nutritional status of the Andorran population 2004–2005**													
Female	6.8	81	164	77			17	75	22	32	10			
Male	8.4	95	197	86			17	84	28	41	13			
***Austria***	**Austrian nutrition report 2010–2012**													
Female	7.5	67	209		43		21	72	31	24	13		1.4	11.6
Male	8.9	79	235		48		21	86	37	28	14		1.5	12.3
***Belgium***	**The Belgian food consumption survey 2014–2015**													
Female	7.9	71	202	94			18	77	28	28	14	0.8		
Male	10.9	95	274	124			20	102	36	37	18	1.0		
***France***	**INCA2 2006–2007**													
Female	7.6	74	199	89			16	80	32	29	12			
Male	9.8	100	262	101			19	100	41	36	15			
***Germany***	**German National Nutrition Survey II 2005–2007**													
Female	7.9	67	227				25	74						
Male	10.5	89	279				27	100						
***Ireland***	**National adult nutrition survey 2008–2010**													
Female	7.1	70	198	81			18	66	29	27	14	1.0	1.6	
Male	9.8	98	260	100			21	90	38	35	16	1.6	1.9	
***Italy***	**The third Italian National food consumption survey INRAN-SCAI 2005–2006**													
Female	8.0	75	236	79			18	77	24	37	10			
Male	9.9	92	282	85			20	94	29	46	12			
***The Netherlands***	**Dutch National Food Consumption Survey (DNFCS) 2007–2010**													
Female	8.2	75	220	106			19	76	29	26	14	1.3	1.7	11.8
Male	11.1	98	291	128			23	103	38	36	20	1.6	2.2	17.0
***Portugal***	**National Food and Physical Activity Survey (IAN-AF) 2015–2016**													
Female	7.2	78	195	77			17	60	22	25	11	0.8		9.5
Male	9.8	106	246	85			20	77	27	32	13	1.0		12.3
***Spain*** **	**ENIDE 2011**													
Female	9.2	88	199	72			19	93	26	39	13			
Male	9.8	109	242	76			21	115	33	48	15			
***UK***	**National Diet and Nutrition Survey (NDNS) Y1-4 2008–2012**													
Female	6.7	65	195	85			13	60	22	21	10	1.1	1.8	8.6
Male	8.7	83	247	105			15	77	28	28	13	1.5	2.2	11.0
**WEST TOTAL Female**	7.8	73	212	84	43		19	75	26	30	12	1.1	1.7	9.5
**WEST TOTAL Male**	9.8	94	264	96	48		21	96	33	38	14	1.4	2.1	12.2
**EUROPE TOTAL Female**	7.6	69	209	84	41		19	73	25	28	13	1.1	1.5	11.9
**EUROPE TOTAL Male**	9.7	90	264	96	48		21	94	32	36	16	1.4	1.9	14.9

* For each country weighted means were calculated for each nutrient by multiplying the male/female mean for each age group by the number of men/women in that age group, then dividing the total by the total number of men/women in the country in question. For each nutrient regional weighted means were calculated by multiplying the male/female mean for each country by the total national population [16], adding this figure for each country and dividing by the total sum of the national populations in that region. For each nutrient total European weighted means were calculated by multiplying the male/female mean for each age country by the total national population [16], adding this figure for each country and dividing by the total sum of the national populations in all three European regions. ** Figures for Spain are based on median rather than mean values.

**Table 5:** Weighted means* by country for micronutrient in 21 national dietary surveys in the WHO Europe region.

SURVEY	Folic Acid (μg)	Vitamin B12 (μg)	Vitamin D (μg)	Calcium (mg)	Potassium (mg)	Sodium (mg)	Iron (mg)	Iodine (μg)	Zinc (mg)
***Estonia***	**National Dietary Survey 2014–2015**								
Female	166	5.8	4.3	648	3037	1801	10.8	108	8.4
Male	198	8.0	5.7	767	3761	2562	13.6	134	11.4
***Hungary***	**Hungarian Dietary Survey 2009**								
Female	131	2.8	2.0	651	2600	5086	9.5		7.5
Male	161	3.7	2.6	701	3140	7100	12.5		10.2
***Latvia***	**Latvian National Food Consumption Survey 2007–2009**								
Female	214	3.7	1.9	457	2250	2283	9.1	53	7.2
Male	214	3.7	1.9	555	2868	3598	12.1	68	10.1
***Lithuania***	**Study and Evaluation of Actual Nutrition and Nutrition Habits of Lithuanian Adult Population 2013–2014**								
Female	366	1.0	3.1	506	2322	2348	8.9	28	7.0
Male	643	1.5	3.7	576	2887	2538	12.2	33	9.6
***Turkey***	**Turkey Nutrition and Health Survey 2010 (TNHS)**								
Female	320	2.5	0.8	583	2242	1625	10.0	58	8.2
Male	393	4.0	1.2	704	2608	2552	12.3	69	10.7
**CEEC TOTAL Female**	**298**	**2.6**	**1.1**	**586**	**2292**	**2019**	**9.9**	**58**	**8.1**
**CEEC TOTAL Male**	**370**	**3.9**	**1.5**	**698**	**2692**	**3041**	**12.3**	**69**	**10.6**
***Denmark***	**Danish Dietary Habits 2011–2013**								
Female	329	5.6	4.3	1038	3200	3200	10.0	227	10.5
Male	370	8.0	5.3	1188	3900	4400	13.0	268	14.1
***Finland***	**The National FINDIET 2012 Survey**								
Female	231	5.0	8.7	1040	3352	2492	10.0	186	10.2
Male	266	7.0	11.8	1178	4037	3400	12.4	228	12.7
***Iceland***	**The Diet of Icelanders—a National Dietary Survey 2010–2011**								
Female	249	5.5	6.6	820	2632	2600	9.4	142	8.8
Male	304	8.4	9.7	1034	3433	3773	12.5	195	12.4
***Norway***	**Norkost3 2010–2011**								
Female	231	6.0	4.9	811	3374	2510	10.0		
Male	279	8.8	6.7	1038	4263	3558	12.5		
***Sweden***	**Riksmaten 2010–2011 Swedish Adult Dietary Survey**								
Female	252	5.0	6.4	825	2887	2766	9.6		
Male	266	6.0	7.6	945	3410	3591	11.5		
**NORTH TOTAL Female**	**260**	**5.3**	**6.1**	**912**	**3142**	**2751**	**9.8**	**205**	**10.3**
**NORTH TOTAL Male**	**291**	**7.2**	**7.8**	**1064**	**3812**	**3721**	**12.2**	**247**	**13.4**
***Andorra***	**Evaluation of the Nutritional Status of the Andorran Population 2004–2005**								
Female	241	5.4	2.6	790	2867	2495	10.8		8.1
Male	255	7.4	4.1	831	3126	3086	13.3		9.9
***Austria***	**Austrian Nutrition Report 2010–2012**								
Female	206	4.1	2.8	771	2504	3027	10.6	133	9.3
Male	209	4.9	3.9	821	2775	3532	11.4	144	11.0
***Belgium***	**The Belgian Food Consumption Survey 2014–2015**								
Female	190	3.7	3.5	720		2062	8.6	127	
Male	226	5.2	4.2	821		2739	11.1	174	
***France***	**INCA2 2006–07**								
Female	268	5.1	2.4	850	2681	2533	11.5	117	9.1
Male	307	6.5	2.7	984	3287	3447	14.9	136	12.4
***Germany***	**German National Nutrition Survey II 2005–2007**								
Female	285	4.4	3.0	1020	3272	2502	12.4	196	9.5
Male	327	6.4	3.9	1115	3779	3418	15.0	248	12.1
***Ireland***	**National Adult Nutrition Survey 2008–2010**								
Female	342	7.8	4.7	851	2694	2231	13.7		9.2
Male	410	7.2	4.7	1038	3426	3060	15.5		11.6
***Italy***	**The third Italian National Food Consumption Survey INRAN-SCAI 2005–2006**								
Female		5.3	2.2	735	2853		10.3		10.5
Male		6.6	2.6	803	3231		12.7		12.5
***The Netherlands***	**Dutch National Food Consumption Survey (DNFCS) 2007–2010**								
Female	252	4.3	3.1	993	3086	2386	9.9	158	9.5
Male	308	5.5	4.1	1151	3895	3165	11.9	201	12.3
***Portugal***	**National Food and Physical Activity Survey (IAN-AF) 2015–2016**								
Female	248	4.7	3.5	730	2999	2647	10.8		9.2
Male	281	5.5	4.0	816	3845	3605	14.0		11.9
***Spain*** **	**ENIDE 2011**								
Female	266	6.1	3.7	835	2865	2347	13.7	85	8.7
Male	296	7.9	4.3	884	3049	2702	16.1	100	10.4
***UK***	**National Diet and Nutrition Survey (NDNS) Y1-4 2008–2012**								
Female	231	4.8	2.7	743	2558	2148	9.6	146	7.6
Male	289	6.1	3.3	896	3044	2793	11.6	187	9.6
**WEST TOTAL Female**	259	5.0	2.8	846	2869	2405	11.3	143	9.1
**WEST TOTAL Male**	302	6.5	3.5	951	3349	3153	13.8	178	11.5
**EUROPE TOTAL Female**	268	4.5	2.7	799	2771	2341	10.9	127	8.9
**EUROPE TOTAL Male**	316	6.0	3.3	908	3245	3163	13.4	156	11.4

* For each country, weighted means were calculated for each nutrient by multiplying the male/female mean for each age group by the number of men/women in that age group, then dividing the total by the total number of men/women in the country in question. For each nutrient regional weighted means were calculated by multiplying the male/female mean for each country by the total national population [16], adding this figure for each country and dividing by the total sum of the national populations in that region. For each nutrient total European weighted means were calculated by multiplying the male/female mean for each age country by the total national population [16], adding this figure for each country and dividing by the total sum of the national populations in all three European regions. ** Figures for Spain are based on median rather than mean values.

## Appendix A. Mean Macronutrient Intakes across Dietary Surveys

**COUNTRY**	**SURVEY**	**YEAR**	**Energy (MJ)**	**Energy (Kcal)**	**Protein (g)**	**CHO (g)**	**Sugars (g)**	**Sucrose (g)**	**Starch (g)**	**Fiber (g)**	**Total Fat (g)**	**Saturates (g)**	**MUFA (g)**	**PUFA (g)**	**TFAs (g)**	**n-3 (g)**	**n-6 (g)**
**Andorra**	**Evaluation of the nutritional status of the Andorran population**	2004–2005															
	female: 25–44 y		6.9	1650	83	165	75			15.8	73	23.4	32.5	10.2			
	female: 45–64 y		6.8	1628	81	162	77			17.6	73	22.3	32.8	10.6			
	female: 65–75 y		6.4	1518	71	165	83			21.3	65	18.3	31.2	8.6			
	male: 25–44 y		8.8	2093	100	205	88			16.8	85	30.7	42.7	13.8			
	male: 45–64 y		8.0	1919	90	188	84			17.1	86	26.5	39.3	12.1			
	male: 65–75 y		7.0	1679	83	173	80			18.3	74	20.8	34.9	12.0			
**Austria**	**Austrian nutrition report**	2010–2012															
	female: 18–24 y		8.0	1917	72	225		43		22.0	77	32.0	25.6	14.9		1.5	12.6
	female: 25–50 y		7.8	1854	70	218		46		22.0	74	30.9	24.7	12.4		1.5	12.2
	female: 51–64 y		7.7	1826	64	219		46		22.0	73	30.4	22.3	14.2		1.5	12.0
	female: 65–80 y		7.0	1675	63	188		38		19.0	69	29.8	22.3	13.0		1.4	10.4
	male: 18–24 y		10.1	2403	90	282		60		24.0	91	37.4	29.4	16.0		1.6	13.9
	male: 25–50 y		9.1	2172	81	239		54		20.0	89	38.6	29.0	14.5		1.5	12.5
	male: 51–64 y		9.4	2245	84	236		45		22.0	92	39.9	29.9	15.0		1.5	13.0
	male: 65–80 y		8.0	1920	67	216		38		20.0	75	32.0	23.5	12.8		1.4	11.1
**Belgium**	**The Belgian food consumption survey 2014–2015**	2014–2015															
	female: 18–39 y		8.2	1955	71	214	99		116	17.3	79	29.0	29.0	14.0	0.8		
	female: 40–64 y		7.6	1826	71	190	89		100	18.8	75	27.0	26.0	14.0	0.8		
	male: 18–39 y		11.1	2652	95	291	131		155	19.3	100	36.0	38.0	18.0	1.0		
	male: 40–64 y		10.7	2547	96	253	115		137	20.1	104	37.0	36.0	19.0	1.1		
**Denmark**	**Danish Dietary habits 2011–2013**	2011–2013															
	female: 18–75 y		8.4	2008	76	211		43		21.0	83	33.0	31.0	13.0	1.3		
	male: 18–75 y		11.2	2677	101	269		56		24.0	111	45.0	41.0	17.0	1.7		
**Estonia**	**National Dietary Survey**	2014–2015															
	female: 18–24 y		6.8	1625	64	200		48		15.1	64	25.5	22.8	10.9	0.5	1.6	8.6
	female: 25–29 y		7.6	1818	71	217		54		17.1	76	30.5	27.2	12.1	0.6	1.9	9.2
	female: 30–34 y		7.3	1762	71	210		49		18.1	73	29.6	26.4	11.6	0.6	1.8	8.9
	female: 35–39 y		7.2	1730	68	205		48		17.8	72	29.1	26.4	11.9	0.6	2.2	9.1
	female: 40–44 y		6.4	1529	60	188		41		17.3	61	24.4	21.8	10.4	0.5	1.8	7.7
	female: 45–49 y		6.2	1488	59	177		38		16.6	60	23.6	21.9	10.3	0.5	1.8	7.7
	female: 50–54 y		6.3	1505	60	183		42		17.6	61	23.3	22.3	10.8	0.5	1.8	7.8
	female: 55–59 y		6.4	1537	64	185		38		18.1	62	24.6	22.6	10.7	0.5	1.5	8.0
	female: 60–64 y		6.2	1474	61	179		39		17.0	59	22.5	22	10.2	0.5	1.7	7.6
	female: 65–69 y		6.3	1509	62	186		36		17.8	59	23.3	21.4	10.3	0.5	1.9	7.5
	female: 70–74 y		5.5	1330	55	168		32		16.6	51	20.3	18.3	8.9	0.4	1.5	6.7
	male: 18–24 y		9.7	2326	102	266		58		18.1	92	35.8	34.4	16.4	0.6	2.8	13.2
	male: 25–29 y		9.5	2277	94	239		55		16.8	93	36.9	35.2	14.7	0.8	3.5	11.4
	male: 30–34 y		8.6	2058	85	234		52		17.7	84	33	31.3	14.4	0.7	2.6	11.2
	male: 35–39 y		9.5	2279	94	252		55		19.7	94	36.9	34.7	16.8	0.6	4.4	12.3
	male: 40–44 y		8.7	2085	89	229		45		19.3	81	31.8	30.9	13.4	0.6	3.0	10.0
	male: 45–49 y		8.6	2068	79	242		47		20.1	80	29.7	31.3	14	0.6	3.6	10.5
	male: 50–54 y		8.9	2125	89	233		46		20.4	89	33.9	33.8	14.9	0.6	4.6	11.1
	male: 55–59 y		8.2	1965	75	221		44		19.2	76	27.6	29	14	0.5	3.5	10.5
	male: 60–64 y		8.1	1941	81	226		40		20.6	75	29.6	28.1	12.6	0.6	2.9	9.3
	male: 65–69 y		7.8	1865	78	213		34		19.5	74	29.8	27.4	12.4	0.6	2.6	9.0
	male: 70–74 y		7.6	1814	75	213		36		19.6	73	29.1	27.5	12.7	0.5	2.4	9.4
**Finland**	**The national FINDIET 2012 survey**	2012															
	female: 25–34 y		7.8	1864	76	199		49		19.0	78	31.0	27.0	13.4	1.0	3.0	9.8
	female: 35–44 y		7.7	1840	77	195		46		20.0	75	29.0	27.0	13.0	0.9	2.9	9.6
	female: 45–54 y		7.0	1673	68	180		44		21.0	67	26.0	24.0	11.6	0.8	2.7	8.4
	female: 55–64 y		6.6	1577	67	171		36		22.0	63	24.0	22.0	11.6	0.7	2.8	8.5
	female: 65–74 y		6.2	1482	62	166		35		21.0	57	22.0	20.0	10.6	0.7	2.5	7.6
	male: 25–34 y		10.2	2449	106	249		55		19.0	102	40.0	37.0	16.9	1.3	3.7	12.5
	male: 35–44 y		9.5	2275	96	237		54		21.0	93	36.0	34.0	15.6	1.1	3.5	11.4
	male: 45–54 y		9.5	2282	96	237		52		23.0	93	36.0	34.0	16.2	1.1	3.6	11.8
	male: 55–64 y		8.6	2053	85	207		45		23.0	86	33.0	30.0	14.9	1.0	3.5	10.8
	male: 65–74 y		8.2	1954	80	212		43		24.0	77	29.0	28.0	13.7	0.9	3.4	9.7
**France**	**INCA2**	2006–2007															
	female: 18–79 y		7.6	1809	74	199	89		105	16.0	80	32.1	28.6	12.3			
	male: 18–79 y		9.8	2348	100	262	101		153	19.2	100	41.2	35.7	14.5			
**Germany**	**German National Nutrition Survey II**	2005–2007															
	female: 19–24 y		8.4	1996	65	252				21.7	74						
	female: 25–34 y		8.5	2031	70	251				24.0	77						
	female: 35–50 y		8.2	1948	69	231				24.7	76						
	female: 51–64 y		7.8	1856	67	217				26.1	72						
	female: 65–80 y		7.3	1753	62	209				24.9	69						
	male: 19–24 y		12.0	2872	102	331				24.6	110						
	male: 325–34 y		11.6	2783	99	318				25.8	110						
	male: 35–50 y		11.0	2640	94	294				27.3	106						
	male: 51–64 y		10.0	2400	86	262				27.4	96						
	male: 65–80 y		9.2	2191	78	241				27.3	88						
**Hungary**	**Hungarian Dietary Survey 2009**	2009															
	female: 19–30 y		9.1	2175	81	265		49		20.7	88	26.2	26.8	22.8		0.9	22.1
	female: 31–60 y		9.0	2151	81	254		44		21.0	88	25.9	27.1	22.7		0.9	22.0
	female: 60+		8.6	2055	75	245		41		20.6	84	25.0	26.3	21.2		0.9	20.4
	male: 19–30 y		12.5	2988	112	334		64		25.5	124	37.5	39.8	30.0		1.2	29.1
	male: 31–60 y		12.3	2940	109	322		49		25.4	127	37.6	41.4	30.4		1.2	29.5
	male: 60+		10.5	2510	92	277		40		23.1	107	31.7	35.1	25.5		1.0	24.6
**Iceland**	**The Diet of Icelanders—a national dietary survey 2010–2011**	2010–2011															
	female: 18–30 y		8.0	1895	75	222	108			16.2	71	27.6	23.2	12.4	1.3	2.6	9.7
	female: 31–60 y		7.5	1795	76	190	86			16.5	74	29.7	23.4	12.5	1.6	3.0	9.4
	female: 61–80 y		6.7	1610	71	161	74			14.8	69	28.4	21.9	10.7	1.6	2.9	7.6
	male: 18–30 y		11.1	2635	116	288	129			19.1	101	38.9	32.5	17.1	2.1	3.5	13.6
	male: 31–60 y		10.1	2402	107	242	105			17.6	101	40.5	32.3	16.2	2.2	3.9	12.3
	male: 61–80 y		8.7	2081	97	192	80			16.7	94	39.1	30.1	13.7	2.3	4.0	9.5
**Ireland**	**National adult nutrition survey**	2008–2010															
	female: 18–64 y		7.2	1721	70	200	81		115	17.3	68	28.9	27.4	13.9	1.1	1.6	
	female: 18–35 y		7.5	1793	69	206	84		117	15.9	70	29.9	29.4	14.8	1.1	1.6	
	female: 36–50 y		7.1	1697	71	197	77		115	17.5	67	28.6	26.4	13.2	1.0	1.6	
	female: 51–64 y		7.0	1673	73	195	83		109	19.5	65	27.9	25.8	13.5	1.0	1.8	
	female: 65+ y		6.5	1554	69	187	80		103	18.4	61	26.5	22.6	11.7	1.0	1.7	
	male: 18–64 y		10.1	2414	100	266	102		160	21.1	92	38.7	36.4	16.9	1.6	2.0	
	male: 18–35 y		10.7	2557	105	281	108		167	21.3	95	39.5	38.3	17.9	1.7	2.0	
	male: 36–50 y		9.7	2318	99	259	98		157	21.0	92	38.6	35.6	16.2	1.5	1.9	
	male: 51–64 y		9.3	2223	93	249	98		148	21.0	86	37.4	33.6	15.9	1.5	2.0	
	male: 65+ y		8.3	1984	85	226	89		133	19.6	78	35.6	29.8	13.1	1.4	1.6	
**Italy**	**The third Italian National food consumption survey INRAN-SCAI**	2005–2006															
	female: 18–64.9		8.1	1939	76	237	80		142	17.7	79	24.4	38.3	10.0			
	female: 65+		7.7	1834	71	234	79		139	18.7	70	22.2	34.1	8.0			
	male: 18–64.9		10.0	2390	93	283	86		179	19.6	95	29.7	45.9	12.2			
	male: 65+		9.6	2296	88	275	82		174	21.6	87	26.8	43.5	10.4			
**Latvia**	**Latvian National Food Consumption Survey 2007–2009**	2007–2009															
	female: ALL		6.7	1613	55	190				15.8	68	28.1	24.0	10.8			
	male: ALL		9.1	2171	79	246				20.2	93	38.1	33.4	14.8			
	female: 17–26 y		7.1	1690													
	female: 27–36 y		6.4	1523													
	female: 37–46 y		6.5	1562													
	female: 47–56 y		6.7	1608													
	female: 57–64 y		6.4	1530													
	male: 17–26 y		10.0	2394													
	male: 27–36 y		10.0	2393													
	male: 37–46 y		9.7	2319													
	male: 47–56 y		9.3	2230													
	male: 57–64 y		8.9	2121													
**Lithuania**	**Study and evaluation of actual nutrition and nutrition habits of Lithuanian adult population**	2013–2014															
	female: 19–75 y		6.5	1561	56	178	56			14.6	71	21.9	26.8	15.5			
	male: 19–75 y		9.2	2188	75	224	55			17.2	108	33.5	41.1	23.8			
	all: 19–34 y		8.1	1936	65	209	58			15.4	92	28.4	34.8	20.1			
	all: 35–49 y		7.8	1855	66	197	56			16.1	90	27.7	34.0	19.7			
	all: 50–64 y		7.4	1763	63	191	55			15.8	83	25.9	31.7	18.3			
	all: 65–75 y		6.7	1600	57	183	51			15.1	72	22.3	27.3	15.8			
**The Netherlands**	**Dutch National Food Consumption Survey (DNFCS) 2007–2010**	2007–2010															
	female: 19–30 y		8.5	2028	73	242	121			18.0	77	29.0	26.9	14.8	1.3	1.5	12.3
	female: 31–50 y		8.3	1983	75	222	104			18.9	77	29.6	26.6	14.6	1.3	1.7	11.9
	female: 51–69 y		7.9	1874	77	195	92			18.8	72	27.8	24.0	13.8	1.4	1.8	11.3
	male: 19–30 y		11.9	2847	98	342	152			22.4	109	39.3	39.1	21.7	1.7	2.3	18.1
	male: 31–50 y		11.1	2651	97	285	126			23.7	104	38.3	36.2	21.0	1.6	2.3	17.4
	male: 51–69 y		10.2	2425	97	246	107			21.6	94	35.4	32.2	18.6	1.6	2.2	15.4
**Norway**	**Norkost3**	2010–2011															
	female: 18–70 y		8.0	1912	81	205		36		22.0	75	29.0	25.0	13.0			
	male: 18–70 y		10.9	2605	112	278		48		26.0	102	39.0	34.0	18.0			
	female: 18–29 y		8.1	1936	80	221		46		21.0	73	28.0	25.0	13.0			
	female: 30–39 y		8.4	2008	83	232		42		24.0	75	29.0	25.0	14.0			
	female: 40–49 y		8.1	1936	83	202		32		22.0	77	30.0	26.0	14.0			
	female: 50–59 y		7.9	1888	81	194		33		22.0	75	28.0	26.0	14.0			
	female: 60–70 y		7.4	1769	77	182		30		22.0	72	28.0	24.0	13.0			
	male: 18–29 y		12.8	3059	130	339		69		29.0	114	44.0	38.0	21.0			
	male: 30–39 y		11.5	2749	118	298		49		26.0	108	42.0	37.0	19.0			
	male: 40–49 y		10.6	2533	107	275		51		25.0	100	38.0	34.0	19.0			
	male: 50–59 y		10.4	2486	109	259		41		26.0	99	37.0	33.0	18.0			
	male: 60–70 y		9.9	2366	102	247		39		27.0	94	36.0	31.0	17.0			
**Portugal**	**National Food and Physical Activity Survey (IAN-AF)**	2015–2016															
	female: 18–64 y		7.3	1747	80	199	78			16.9	61	23.4	25.2	11.1	0.9		9.9
	female: 65–84 y		6.5	1555	70	180	73			18.1	53	17.3	21.7	9.1	0.6		7.9
	male: 18–64 y		10.1	2398	111	255	89			19.9	81	28.9	33.9	13.7	1.1		13.1
	male: 65–84 y		8.5	2030	91	212	71			20.6	63	20.9	26.4	10.8	0.7		9.6
**Spain**	**ENIDE 2011**	2009–2010															
	female: 18–24 y		9.2	2186	88	209				17.1	95	27.5	40.1	13.0			
	female: 25–44 y		9.2	2187	88	202				18.9	94	26.2	38.9	12.4			
	female: 45–64 y		9.1	2162	88	193				19.7	91	24.2	38.1	12.6			
	male: 18–24 y		10.1	2402	117	275				20.5	127	39.6	53.3	17.1			
	male: 25–44 y		9.8	2340	109	248				20.4	117	33.6	49.1	15.7			
	male: 45–64 y		9.6	2281	106	222				21.7	108	29.0	45.1	14.5			
**Sweden**	**Riksmaten 2010–11 Swedish Adult Dietary Survey**	2010–2011															
	female: 18–30 y		7.6	1819	69	205		44		17.3	72	27.4	27.1	12.0		2.4	9.2
	female: 31–44 y		7.6	1820	73	199		38		18.5	72	27.8	26.9	11.6		2.4	8.7
	female: 45–64 y		7.3	1755	73	182		34		19.3	70	26.5	26.0	11.7		2.5	8.7
	female: 65–80 y		7.1	1703	70	186		34		20.0	65	24.9	23.9	10.6		2.6	7.6
	male: 18–30 y		9.4	2246	95	241		45		18.6	88	34.1	32.8	14.0		2.7	10.6
	male: 31–44 y		9.8	2343	95	250		43		21.3	92	35.0	34.9	14.9		2.9	11.4
	male: 45–64 y		9.4	2254	93	237		41		21.8	87	33.7	32.8	13.9		2.9	10.3
	male: 65–80 y		8.7	2083	84	223		38		22.5	80	30.5	29.6	13.4		3.1	9.7
**Turkey**	**Turkey nutrition and health survey 2010 (TNHS)**	2010															
	female: 19–30 y		6.9	1649	52	204				19.0	67	21.7	23.1	17.4		1.2	16.1
	female: 31–50 y		6.9	1638	52	205				20.3	65	21.1	22.4	17.3		1.2	16.0
	female: 51–64 y		6.4	1533	49	195				21.0	59	19.5	21.5	14.2		1.1	13.1
	female: 65–74 y		5.9	1409	46	183				19.3	53	16.8	19.0	13.4		0.9	12.4
	female: 75+ y		5.1	1223	39	156				16.5	47	16.0	17.2	10.7		0.8	9.8
	male: 19–30 y		9.4	2242	71	282				22.4	86	28.3	30.0	21.9		1.6	20.2
	male: 31–50 y		9.2	2203	73	278				23.7	83	27.4	29.3	20.4		1.5	18.8
	male: 51–64 y		8.0	1919	64	242				24.0	72	23.8	26.5	17.1		1.3	15.7
	male: 65–74 y		7.1	1705	56	220				22.9	64	21.5	23.4	15.0		1.1	13.7
	male: 75+ y		6.7	1606	52	207				21.4	61	20.1	24.0	13.0		1.1	11.9
**UK**	**National Diet and Nutrition Survey (NDNS) Years 1–4**	2008–2012															
	female: 19–64		6.8	1613	65	197	85		113	12.8	60	22.1	21.7	10.6	1.1	1.8	8.8
	female: 65+ y		6.4	1510	64	187	88		98	13.1	58	23.0	19.6	9.5	1.2	1.8	7.7
	male: 19–64		8.9	2111	85	251	106		146	14.7	78	28.4	28.5	13.4	1.5	2.2	11.2
	male: 65+ y		8.1	1935	78	231	102		129	14.9	74	28.7	25.8	12.4	1.5	2.3	10.1
	all: 19–64		7.8	1861	75	224	95		129	13.7	69	25.2	25.1	12.0	1.3	2.0	10.0
	all: 65+ y		7.1	1697	70	206	95		112	13.9	65	25.5	22.3	10.7	1.3	2.0	8.7

## Appendix B. Mean Micronutrient Intakes across Dietary Surveys

**COUNTRY**	**SURVEY**	**YEAR**	**Folic Acid (μg)**	**Vitamin B12 (μg)**	**Vitamin D (μg)**	**Calcium (mg)**	**Potassium (mg)**	**Sodium (mg)**	**Iron (mg)**	**Iodine (μg)**	**Zinc (mg)**
**Andorra**	**Evaluation of the nutritional status of the Andorran population**	2004–2005									
	female: 25–44 y		227	5.3	3.4	793	2751	2662	10.8		8.4
	female: 45–64 y		258	5.8	2.0	772	2912	2401	10.9		7.9
	female: 65–75 y		254	4.6	0.7	834	3252	2030	10.5		7.4
	male: 25–44 y		248	7.1	5.1	863	3124	3272	13.2		10.4
	male: 45–64 y		248	8.1	2.9	797	3102	2835	13.4		9.7
	male: 65–75 y		302	7.4	1.5	737	3179	2644	13.8		7.8
**Austria**	**Austrian nutrition report**	2010–2012									
	female: 18–24 y		229	3.6	2.0	956	2562	3000	11.4	161	10.4
	female: 25–50 y		216	4.0	2.8	838	2632	2800	10.9	130	9.7
	female: 51–64 y		193	3.3	2.7	786	2623	2600	10.3	141	9.1
	female: 65–80 y		194	4.8	3.2	632	2288	3480	10.2	124	8.6
	male: 18–24 y		255	5.5	4.0	991	3329	3400	13.9	160	12.4
	male: 25–50 y		197	5.3	3.6	881	2768	3240	11.8	143	11.4
	male: 51–64 y		222	5.0	4.6	802	2820	3320	11.6	142	11.9
	male: 65–80 y		203	4.0	3.9	692	2593	4200	9.9	142	9.2
**Belgium**	**The Belgian food consumption survey 2014–2015**	2014–2015									
	female: 18–39 y		189	3.6	3.4	704		2076	8.5	123	
	female: 40–64 y		191	3.7	3.6	737		2047	8.8	132	
	male: 18–39 y		228	5.0	4.0	842		2731	11.0	171	
	male: 40–64 y		224	5.5	4.6	795		2748	11.4	177	
**Denmark**	**Danish Dietary habits 2011–2013**	2011–2013									
	female: 18–75 y		329	5.6	4.3	1038	3200	3200	10.0	227	10.5
	male: 18–75 y		370	8.0	5.3	1188	3900	4400	13.0	268	14.1
**Estonia**	**National Dietary Survey**	2014–2015									
	female: 18–24 y		159	4.4	3.1	671	2800	1737	9.6	112	7.8
	female: 25–29 y		178	5.8	4.5	729	3200	1890	12.0	123	9.2
	female: 30–34 y		174	5.6	4.6	730	3200	1820	11.9	119	9.3
	female: 35–39 y		172	6.2	4.2	715	3200	1878	11.6	117	9.0
	female: 40–44 y		167	5.5	3.7	620	3100	1847	10.3	107	8.0
	female: 45–49 y		164	5.9	4.6	595	3000	1687	9.6	93	7.7
	female: 50–54 y		175	7.4	5.3	591	3000	1657	10.2	96	7.9
	female: 55–59 y		173	5.7	4.4	614	3100	1718	10.2	102	8.4
	female: 60–64 y		152	5.5	4.3	566	2900	1827	10.0	102	8.0
	female: 65–69 y		156	7.5	4.9	601	3000	1909	12.9	102	8.3
	female: 70–74 y		143	5.0	3.8	545	2700	1700	9.1	85	7.4
	male: 18–24 y		219	6.6	4.3	950	3900	2571	14.8	149	12.1
	male: 25–29 y		210	7.6	5.0	833	3800	2798	13.5	134	11.7
	male: 30–34 y		209	9.1	4.0	788	3800	2412	14.2	132	11.1
	male: 35–39 y		203	7.8	4.7	894	3900	2608	14.3	151	12.4
	male: 40–44 y		194	7.5	5.6	729	3800	2396	13.4	130	11.5
	male: 45–49 y		196	5.9	5.7	685	3900	2416	13.1	125	11.2
	male: 50–54 y		205	10.9	6.4	777	4000	3014	13.7	135	12.0
	male: 55–59 y		186	7.0	7.6	621	3500	2607	12.9	120	10.4
	male: 60–64 y		191	9.7	6.6	652	3700	2580	13.1	121	11.1
	male: 65–69 y		173	7.9	7.9	720	3600	2396	12.2	134	10.4
	male: 70–74 y		182	8.4	6.6	636	3400	2395	13.0	125	10.8
**Finland**	**The national FINDIET 2012 survey**	2012									
	female: 25–34 y		243	5.3	8.2	1206	3500	2600	10.0	190	11.0
	female: 35–44 y		233	5.1	9.0	1155	3400	2700	11.0	190	11.0
	female: 45–54 y		230	4.9	8.2	952	3300	2500	10.0	190	10.0
	female: 55–64 y		233	4.7	9.1	1002	3400	2500	10.0	190	10.0
	female: 65–74 y		219	5.1	8.7	921	3200	2200	9.0	173	9.0
	male: 25–34 y		277	6.9	10.7	1424	4200	3700	12.0	235	14.0
	male: 35–44 y		272	7.3	11.5	1251	4100	3400	13.0	235	13.0
	male: 45–54 y		277	8.0	11.2	1195	4200	3700	13.0	235	13.0
	male: 55–64 y		257	6.4	11.9	1099	3900	3300	12.0	235	12.0
	male: 65–74 y		255	6.7	12.8	1056	3900	3100	12.0	209	12.0
**France**	**INCA2**	2006–2007									
	female: 18–79 y		268	5.1	2.4	850	2681	2533	11.5	117	9.1
	male: 18–79 y		307	6.5	2.7	984	3287	3447	14.9	136	12.4
**Germany**	**German National Nutrition Survey II**	2005–2007									
	female: 19–24 y		318	4.0	2.0	1039	2997	2355	11.6	173	9.1
	female: 25–34 y		311	4.4	2.6	1061	3260	2533	12.6	192	9.8
	female: 35–50 y		285	4.4	2.7	1067	3331	2579	12.8	200	9.8
	female: 51–64 y		281	4.6	3.4	1011	3391	2522	12.6	204	9.6
	female: 65–80 y		264	4.3	3.4	918	3125	2376	11.4	190	8.8
	male: 19–24 y		394	6.9	3.0	1281	3812	3739	15.6	257	13.2
	male: 325–34 y		372	6.9	3.5	1252	3890	3620	15.9	255	13.2
	male: 35–50 y		337	6.5	3.8	1167	3939	3582	15.7	256	12.7
	male: 51–64 y		316	6.4	4.2	1071	3769	3346	14.7	246	11.7
	male: 65–80 y		282	5.9	4.4	970	3498	3058	13.6	232	10.9
**Hungary**	**Hungarian Dietary Survey 2009**	2009									
	female: 19–30 y		130	3.1	2.0	691	2600	5000	9.6		7.9
	female: 31–60 y		133	2.9	2.0	647	2600	5200	9.7		7.7
	female: 60+		129	2.6	1.9	636	2600	4900	9.2		7.0
	male: 19–30 y		167	3.6	2.8	772	3200	7100	12.8		10.6
	male: 31–60 y		166	3.9	2.7	698	3200	7400	12.9		10.5
	male: 60+		142	3.0	2.3	635	2900	6200	11.1		8.8
**Iceland**	**The Diet of Icelanders—a national dietary survey 2010–2011**	2010–2011									
	female: 18–30 y		270	4.6	4.6	930	2543	2677	10.3	116	9.4
	female: 31–60 y		259	5.3	6.4	840	2708	2631	9.7	138	9.1
	female: 61–80 y		209	6.6	8.6	694	2517	2474	8.2	168	7.8
	male: 18–30 y		343	7.5	6.6	1215	3429	4057	13.3	169	13.9
	male: 31–60 y		309	7.7	9.3	1047	3489	3775	12.9	200	12.4
	male: 61–80 y		258	10.8	13.4	847	3308	3520	11.0	204	11.2
**Ireland**	**National adult nutrition survey**	2008–2010									
	female: 18–64 y		339	8.0	3.9	824	2690	2268	13.7		9.0
	female: 18–35 y		337	11.1	3.1	794	2507	2385	15.1		8.5
	female: 36–50 y		301	5.4	3.5	824	2781	2220	12.8		8.7
	female: 51–64 y		399	6.9	6.0	874	2855	2145	12.8		10.1
	female: 65+ y		357	6.5	8.5	995	2721	2035	13.8		10.7
	male: 18–64 y		407	7.3	4.6	1060	3491	3122	15.1		11.8
	male: 18–35 y		426	7.4	3.9	1122	3568	3291	15.6		12.4
	male: 36–50 y		383	7.4	4.7	1036	3463	3123	14.8		11.6
	male: 51–64 y		404	7.2	5.7	981	3388	2817	14.3		11.2
	male: 65+ y		427	6.4	5.2	908	3038	2689	18.1		10.2
**Italy**	**The third Italian National food consumption survey INRAN-SCAI**	2005–2006									
	female: 18–64.9			5.5	2.3	730	2861		10.4		10.6
	female: 65+			4.4	1.8	754	2822		10.0		9.9
	male: 18–64.9			6.6	2.6	799	3218		12.6		12.6
	male: 65+			6.5	2.5	825	3300		13.2		12.2
**Latvia**	**Latvian National Food Consumption Survey 2007–2009**	2007–2009									
	female: ALL					457	2250		9.1	53	7.2
	male: ALL					555	2868		12.1	68	10.1
	female: 17–26 y		218	3.3	1.4			2240			
	female: 27–36 y		214	3.6	1.6			1920			
	female: 37–46 y		213	3.9	1.9			2640			
	female: 47–56 y		212	3.7	2.5			2320			
	female: 57–64 y		208	4.5	2.2			2160			
	male: 17–26 y		218	3.3	1.4			3480			
	male: 27–36 y		214	3.6	1.6			3960			
	male: 37–46 y		213	3.9	1.9			3680			
	male: 47–56 y		212	3.7	2.5			3400			
	male: 57–64 y		208	4.5	2.2			3600			
**Lithuania**	**Study and evaluation of actual nutrition and nutrition habits of Lithuanian adult population**	2013–2014									
	female: 19–75 y		481	1.2	3.4	535	2556	2842	10.3	30	8.1
	male: 19–75 y		366	1.0	3.1	506	2322	2348	8.9	28	7.0
	all: 19–34 y		643	1.5	3.7	576	2887	2538	12.2	33	9.6
	all: 35–49 y		350	1.4	3.2	575	2654	245	10.7	30	8.6
	all: 50–64 y		459	1.0	1.5	531	2625	2935	10.7	32	8.3
	all: 65–75 y		669	1.2	4.9	518	2519	2882	10.0	30	7.7
**The Netherlands**	**Dutch National Food Consumption Survey (DNFCS) 2007–2010**	2007–2010									
	female: 19–30 y		232	3.9	2.8	954	2847	2429	9.3	156	9.2
	female: 31–50 y		243	4.3	3.1	993	3112	2428	10.1	158	9.5
	female: 51–69 y		281	4.8	3.5	1031	3296	2301	10.4	160	9.9
	male: 19–30 y		293	5.3	3.9	1133	3774	3394	11.6	210	12.0
	male: 31–50 y		302	5.4	3.9	1171	4048	3177	12.4	202	12.5
	male: 51–69 y		330	5.8	4.4	1149	3866	2920	11.8	192	12.3
**Norway**	**Norkost3**	2010–2011									
	female: 18–70 y		231	6.0	4.9	811	3400	2500	9.9		
	male: 18–70 y		279	8.9	6.7	1038	4200	3600	13.0		
	female: 18–29 y		219	5.7	3.9	834	3100	2500	9.4		
	female: 30–39 y		247	5.3	4.3	836	3400	2600	11.0		
	female: 40–49 y		231	6.1	5.0	828	3400	2600	10.0		
	female: 50–59 y		233	6.4	5.2	784	3500	2500	10.0		
	female: 60–70 y		224	6.4	5.8	768	3400	2300	9.3		
	male: 18–29 y		314	8.9	5.5	1248	4300	4000	14.0		
	male: 30–39 y		295	8.9	6.1	1202	4200	4000	13.0		
	male: 40–49 y		257	8.4	6.0	1009	4200	3500	12.0		
	male: 50–59 y		275	8.9	7.3	955	4300	3500	12.0		
	male: 60–70 y		269	9.1	7.8	900	4300	3100	12.0		
**Portugal**	**National Food and Physical Activity Survey (IAN-AF)**	2015–2016									
	female: 18–64 y		245.7	4.8	3.5	731	2990	2690	10.9		9.4
	female: 65–84 y		260.1	4.2	3.5	724	3044	2449	10.3		8.3
	male: 18–64 y		285.7	5.7	4.1	830	3901	3700	14.2		12.4
	male: 65–84 y		264.6	4.8	3.8	764	3639	3260	13.4		10.2
**Spain**	**ENIDE 2011**	2009–2010									
	female: 18–24 y		234	5.2	3.2	789	2590	2328	12.5	75	8.6
	female: 25–44 y		265	5.8	3.5	851	2838	2420	14.1	87	8.8
	female: 45–64 y		281	6.7	4.0	839	3007	2283	13.8	87	8.7
	male: 18–24 y		287	7.7	4.1	958	2905	2756	15.9	95	11.2
	male: 25–44 y		288	7.9	4.3	898	2998	2730	16.1	100	10.4
	male: 45–64 y		309	8.1	4.3	840	3160	2652	16.2	103	10.1
**Sweden**	**Riksmaten 2010–11 Swedish Adult Dietary Survey**	2010–2011									
	female: 18–30 y		223	4.0	5.2	806	2659	2767	8.9		9.2
	female: 31–44 y		247	4.8	6.2	849	2865	2876	9.7		9.9
	female: 45–64 y		263	5.0	6.6	805	2971	2755	9.9		9.7
	female: 65–80 y		275	6.4	7.6	826	3013	2546	9.4		9.1
	male: 18–30 y		244	5.8	6.6	975	3139	3649	10.8		12.6
	male: 31–44 y		263	5.5	6.9	991	3433	3819	11.7		13.0
	male: 45–64 y		271	6.1	7.7	937	3523	3638	11.9		12.6
	male: 65–80 y		279	6.6	9.1	885	3392	3214	11.0		10.9
**Turkey**	**Turkey nutrition and health survey 2010 (TNHS)**	2010									
	female: 19–30 y		308	3.1	0.9	566	2211	1596	9.9	57	8.4
	female: 31–50 y		334	2.7	0.9	605	2311	1686	10.4	60	8.6
	female: 51–64 y		335	2.3	0.7	606	2357	1636	10.3	59	8.2
	female: 65–74 y		296	2.0	0.5	547	2063	1572	9.5	53	7.6
	female: 75+ y		271	2.0	1.0	495	1855	1426	8.1	49	6.3
	male: 19–30 y		385	4.4	1.1	676	2511	2411	12.4	67	11.2
	male: 31–50 y		410	4.7	1.3	744	2717	2353	13.0	74	11.5
	male: 51–64 y		400	3.7	1.3	713	2687	2197	12.2	68	10.3
	male: 65–74 y		375	2.8	1.2	677	2537	1938	11.1	64	9.2
	male: 75+ y		329	2.3	0.6	593	2192	1811	10.2	55	8.4
**UK**	**National Diet and Nutrition Survey (NDNS) Years 1–4**	2008–2012									
	female: 19–64		228	4.6	2.6	728	2532	1995	9.6	140	7.6
	female: 65+ y		241	5.5	2.9	796	2649	2680	9.4	169	7.6
	male: 19–64		287	5.7	3.1	888	3039	2600	11.7	180	9.7
	male: 65+ y		295	7.6	3.9	924	3063	3480	11.1	213	9.2
	all: 19–64		258	5.1	2.8	807	2785	2297	10.7	160	8.6
	all: 65+ y		265	6.4	3.3	852	2831	3040	10.2	188	8.3
